# Sparser and Less Efficient Hippocampal-Prefrontal Projections account for Developmental Network Dysfunction in a Model of Psychiatric Risk Mediated by Gene-Environment Interaction

**DOI:** 10.1523/JNEUROSCI.1203-21.2021

**Published:** 2022-01-26

**Authors:** Lingzhen Song, Xiaxia Xu, Peggy Putthoff, David Fleck, Marc Spehr, Ileana L. Hanganu-Opatz

**Affiliations:** ^1^Institute of Developmental Neurophysiology, Center for Molecular Neurobiology, University Medical Center Hamburg-Eppendorf, Hamburg 20251, Germany; ^2^Department of Chemosensation, Institute for Biology II, Rheinisch-Westfälische Technische Hochschule Aachen (RWTH) Aachen University, Aachen 52074, Germany

**Keywords:** axonal projections, development, hippocampus, mouse model of psychiatric risk, network oscillations, prefrontal cortex

## Abstract

Precise information flow from the hippocampus (HP) to prefrontal cortex (PFC) emerges during early development and accounts for cognitive processing throughout life. On flip side, this flow is selectively impaired in mental illness. In mouse models of psychiatric risk mediated by gene-environment interaction (GE), the prefrontal-hippocampal coupling is disrupted already shortly after birth. While this impairment relates to local miswiring in PFC and HP, it might be also because of abnormal connectivity between the two brain areas. Here, we test this hypothesis by combining *in vivo* electrophysiology and optogenetics with in-depth tracing of projections and monitor the morphology and function of hippocampal afferents in the PFC of control and GE mice of either sex throughout development. We show that projections from the hippocampal CA1 area preferentially target layer 5/6 pyramidal neurons and interneurons, and to a lesser extent layer 2/3 neurons of prelimbic cortex (PL), a subdivision of PFC. In neonatal GE mice, sparser axonal projections from CA1 pyramidal neurons with decreased release probability reach the PL. Their ability to entrain layer 5/6 oscillatory activity and firing is decreased. These structural and functional deficits of hippocampal-prelimbic connectivity persist, yet are less prominent in prejuvenile GE mice. Thus, besides local dysfunction of HP and PL, weaker connectivity between the two brain areas is present in GE mice throughout development.

**SIGNIFICANCE STATEMENT** Poor cognitive performance in mental disorders comes along with prefrontal-hippocampal dysfunction. Recent data from mice that model the psychiatric risk mediated by gene-environment (GE) interaction identified the origin of deficits during early development, when the local circuits in both areas are compromised. Here, we show that sparser and less efficient connectivity as well as cellular dysfunction are the substrate of the weaker excitatory drive from hippocampus (HP) to prefrontal cortex (PFC) as well as of poorer oscillatory coupling between the two brain areas in these mice. While the structural and functional connectivity deficits persist during the entire development, their magnitude decreases with age. The results add experimental evidence for the developmental miswiring hypothesis of psychiatric disorders.

## Introduction

The brain circuitry accounting for memory and executive abilities in mammals is highly complex and extends over cortical and subcortical areas, yet two brain areas, hippocampus (HP) and prefrontal cortex (PFC), are considered as being its core (Bähner and Meyer-Lindenberg, 2017). Both areas are involved in memory processing: the HP controls episodic memory, whereas the PFC allows adaptative flexibility of memory processing (Spellman et al., 2015; Eichenbaum, 2017). The HP and PFC tightly interact to achieve memory retrieval and consolidation (Miller and Cohen, 2001; Preston and Eichenbaum, 2013) via direct monosynaptic as well as indirect polysynaptic projections (Jay et al., 1989; Dolleman-Van Der Weel and Witter, 1996; Vertes, 2006). The highly efficient communication relevant for memory processing is mediated by oscillatory synchrony of neural activity in the two brain areas (Siapas et al., 2005; Backus et al., 2016; Alemany-González et al., 2020). On the flip side, abnormal episodic memory, as one trait of a broader pattern of deficits in higher cognitive functions, has been reported for psychiatric disorders (Dere et al., 2010; Greenland-White et al., 2017). The cognitive impairment significantly contributes to disability and represents a major burden for patients because it is generally treatment refractory (Bora et al., 2010). Poor cognitive performance relates to reduced prefrontal-hippocampal connectivity in both schizophrenia patients, prodromal and high-risk subjects as well as mouse models of disease (Friston and Frith, 1995; Meyer-Lindenberg et al., 2005; Sigurdsson et al., 2010; Greenland-White et al., 2017). In line with the neurodevelopmental origin of schizophrenia, it has been hypothesized that, while the behavioral symptoms are firstly detectable in young adulthood, the underlying network is compromised at a much earlier stage (Owen et al., 2016).

Experimental confirmation of this hypothesis in human subjects faces major technical and ethical limitations. Therefore, animal models, despite being able to mimic only some disease features (e.g., etiology, neurochemical deficits, behavioral symptoms), are instrumental for uncovering the mechanisms of mental illness-related dysfunction (Nestler and Hyman, 2010; Sigurdsson, 2016; Diamantopoulou and Gogos, 2019). Genetic models, such as mice modeling 22q11.2 microdeletions identified in patients (McDonald-McGinn et al., 2015) as well as models combining genetic deficits and environmental stressors related to higher disease risk [dual-hit genetic-environmental (GE) models; Kannan et al., 2013] show cognitive impairment and abnormal communication within prefrontal-hippocampal circuits (Sigurdsson et al., 2010). We previously showed that these deficits emerge already early in life, at a developmental stage corresponding to neonatal period in mice (first postnatal week) and third gestational trimester in humans (Clancy et al., 2001). Dual-hit GE mice mimicking both the genetic [mutation of the intracellular hub of developmental processes disrupted-in-schizophrenia 1 (DISC1) gene; Brandon and Sawa, 2011] and the environmental [challenge by maternal immune activation (MIA)] background that has been related to mental illness, have abnormal patterns of early electrical activity both in PFC and HP (Hartung et al., 2016; Xu et al., 2019, 2021; Chini et al., 2020). Additionally, prefrontal-hippocampal coupling through synchrony of oscillatory activity as well as directed HP-to-PFC interactions are diminished. Three mechanisms might cause these early deficits: (1) local disruption of prefrontal circuits, (2) local disruption of hippocampal circuits, and/or (3) abnormal long-range communication between PFC and HP. We previously confirmed the first two mechanisms and reported that (1) layer 2/3 pyramidal neurons in PFC experienced excessive microglia-induced synaptic pruning leading to impaired β-γ oscillations (Chini et al., 2020) and (2) the sharp-waves, firing, and network activity in hippocampal CA1 area are decreased in GE mice (Xu et al., 2021). Here, we address the third hypothesis and investigate the long-range connectivity between HP and PFC throughout development in dual-hit GE mice. We show that both structural and functional deficits of hippocampal innervation of PFC compromise the communication between the two brain areas.

## Materials and Methods

### Animals

All experiments were performed in compliance with the German laws and the guidelines of the European Community for the use of animals in research and were approved by the local ethical committee (G17/015, N18/015). Timed-pregnant mice from the University Medical Center Hamburg-Eppendorf animal facility were housed individually at a 12/12 h light/dark cycle and were given access to water and food *ad libitum*. The day of vaginal plug detection was considered embryonic day (E) 0.5, the day of birth was considered postnatal day (P)0. The heterozygous offspring carrying a DISC1 allele (DISC1^Tm1Kara^) on a C57BL/6J background, whose dams were injected at E9.5 with the viral mimetic polyinosinic-polycytidylic acid (poly I:C, 4 mg/kg, i.p.), were classified as dual-hit genetic-environmental (GE) mice (Hartung et al., 2016). Pups born from homozygous Disc1^Tm1Kara^ dams and wild-type males, and pups born from wild-type dams and homozygous Disc1^Tm1Kara^ males were pooled together, as no difference between the two groups was found. Genotypes were assessed using genomic DNA (tail biopsies) and the following primer sequences: forward primer 5′-TAGCCACTCTCATTGTCAGC-3′ and reverse primer 5′-CCTCATCCCTTCCACTCAGC-3′. Nontreated wild-type C57BL/6J mice and the offspring of dams injected at E9.5 with saline (0.9%) were used as controls (CON) and combined together, as no difference between the two groups was found. All experiments were performed on pups of both sexes during neonatal development at P8–P10, as well as during prejuvenile development at P20–P24.

### Stereotaxic injections

The pups were placed in a stereotactic apparatus and kept under anesthesia with isoflurane (induction: 5%, maintenance: 2.5%) for the entire procedure. For retrograde tracing, fluorogold (FG; 2.5%, Fluorochrome, LLC) was iontophoretically injected into the PFC (0.5 mm anterior to bregma, 0.3 mm right to the midline) of P7 or P21 mice. For anterograde tracing, biotinylated dextran amine [BDA; 5% in 0.125 m phosphate buffer (PB), Thermo Fisher Scientific] was iontophoretically injected in the HP (0.7 mm anterior to λ, 2.3 mm right to the midline) of P7 or P21 mice. A glass capillary (∼25 mm tip diameter) was filled with ether ∼1-µl FG or ∼1-µl BDA by capillary forces, and a silver wire was inserted such that it was in contact with the FG or BDA solution. For both anterograde and retrograde tracing, the positive pole of iontophoresis device was attached to the silver wire, whereas the negative one was attached to the skin of the neck. The capillary was carefully lowered into the PFC (∼1.5 mm dorsal from the dura) or HP (∼1.5 mm dorsal from the dura). For injections, anodal current to the pipette (6 s on/off current pulses of 6 mA) was applied for 10 min. For recordings of PL-projecting cells in CA1, a 0.5-µl syringe (Hamilton Company) was attached to a microsyringe pump controller (Micro4, WPI) and Alexa Fluor 555-conjugated cholera toxin subunit B (CTB; 1.0 mg/ml, 150 nl, 80 µl/min, Thermo Fisher Scientific) was injected into the PL of P6 mice at the same coordinates as for FG injection.

For transsynaptic labeling, a 0.5-µl syringe was attached to a microsyringe pump controller and wheat germ agglutinin (WGA; 200 nl 4%, Vector Laboratories) was injected at a rate of 80 µl/min into the HP at the same coordinates as for BDA injection. For all optogenetic experiments, the same procedure was used to inject AAV9-hSyn-hChR2(H134R)-EYFP (Addgene, 2.67 × 10^13^ GC/µl) into the HP (80 nl, 50 nl/min for P1 mice and 150 nl, 80 nl/min for P13–P15 mice). Following injection, the pipette or syringe was left in place for at least 5 min to allow optimal diffusion of the solution. The scalp was closed by application of tissue adhesive glue. The pups were warmed on a heating pad for 10–15 min and returned to the dam until full recovery of the motor activity. The pups were perfused 3 d later for FG and BDA staining. The perfusion occurred 30 h after WGA injection, in line with literature and our pilot data that showed transsynaptic transfer to the first order but no other downstream neurons (Phillips et al., 2019). All the WGA-positive cells were costained with NeuN to exclude the possibility of non-neuronal innervation (data not shown).

### *In utero* electroporation and clearing

Timed-pregnant CON or GE mice (E15.5) were injected subcutaneously with buprenorphine (0.05 mg/kg body weight) 30 min before surgery. Surgery was performed on a heating blanket, and toe pinch and breathing were monitored throughout. Under isoflurane anesthesia (induction: 5%, maintenance: 3.5%), the eyes of the dam were covered with eye ointment to prevent damage before the uterine horns were exposed and moistened with warm sterile PBS (37°C). Solution containing 1.25 µg/µl pAAV-CAG-tDimer2 and 0.1% fast green dye at a volume of 0.75–1.25 µl was injected into the right lateral ventricle of individual embryos using pulled borosilicate glass capillaries with a sharp and long tip. Plasmid DNA was purified with NucleoBond (Macherey-Nagel). To target intermediate and ventral HP (i/vHP), a tri-polar approach was used (Szczurkowska et al., 2016). Each embryo within the uterus was placed between the electroporation tweezer-type paddles (5 mm diameter, both positive poles, Protech) that were oriented at a 90° leftward angle from the midline and a 0° angle downward from anterior to posterior. A third custom build negative pole was positioned on top of the head roughly between the eyes. Electrode pulses (30 V, 50 ms) were applied six times at intervals of 950 ms controlled by an electroporator (CU21EX, BEX). Uterine horns were placed back into the abdominal cavity after electroporation. The abdominal cavity was filled with warm sterile PBS (37°C) and abdominal muscles and skin were sutured individually with absorbable and non-absorbable suture thread, respectively. After recovery, pregnant mice were returned to their home cages, which were half placed on a heating blanket for 2 d after surgery, and received on a daily basis additional wet food supplemented with two to four drops of Metacam (0.5 mg/ml, Boehringer-Ingelheim).

Fluorescence expression was confirmed at P2 using a portable fluorescence flashlight. At P10, pups were anesthetized with 10% ketamine (aniMedica)/2% xylazine (WDT) in 0.9% NaCl solution (10 µg/g body weight, intraperitoneally) and transcardially perfused with Histofix (Carl Roth) containing 4% paraformaldehyde. Brain clearing was performed as previously described (Chung and Deisseroth, 2013). Brains were postfixed overnight at 4°C to maintain structural integrity in hydrogel fixation solution containing 4% acrylamide, 0.05% bis-acrylamide, 0.25% VA-044 Initiator, 4% PFA in PBS^−/−^. To allow hydrogel polymerization, oxygen was removed via a vacuum pump connected to a desiccator. Argon was released and removed twice to establish O_2_-free conditions. After heat-triggered polymerization (37°C; 3 h), samples were extracted from hydrogel and washed in a clearing solution containing 200 mm boric acid and 138 mm SDS (pH 8.5) for 24 h at room temperature (RT). Embedded brains were cleared at 37°C for 48 d. Clearing solution was changed twice each week. DRAQ5 (nuclear marker; 1:1000) was added for 2 d. Next, removal of SDS [washing (3×) in PBST (0.1% Triton X-100 in PBS^−/−^) at RT] terminated clearing. Imaging was performed after 24-h incubation in RIMS80 containing 80 g Nycodenz, 20 mm PS, 0.1% Tween 20, and 0.01% sodium acid. Imaging was performed with a Cleared tissue LightSheet (Intelligent Imaging Innovations) dual-side illumination lightsheet microscope for whole organ imaging, equipped with a PlanNeoFluar 1.0×/0.25 NA objective. 3D stacks were acquired sequentially. A 640-nm laser was used for excitation of DRAQ5 using a multi-line Set 43HE filter cube and a 561-nm laser for tDimer Ds-Red Filter cube. Image stacks were stitched using slidebook 6 software. Brain regions of interest were manually marked using nuclear staining by inspecting coronal slices of the 3D dataset using Imaris 9.7. Fibers were reconstructed and fiber volume within the prelimbic (PL) cortex was calculated. The fiber volume/PL volume ratio was normalized to the transfected cell count in the lateral hippocampal region.

### Electrophysiological recordings and optogenetic manipulation *in vivo*

Multisite extracellular recordings were performed in the PL subdivision of the PFC from P8 to P10 or P20–P24 mice. For recordings in non-anesthetized state in P8–P10 mice, 0.5% bupivacaine/1% lidocaine was locally applied on the neck muscles. For recordings in anesthetized state in P20–P24 mice, mice were injected intraperitoneally with urethane (1 mg/g body weight; Sigma-Aldrich) before surgery. For both groups, the surgery was performed under isoflurane anesthesia (induction: 5%; maintenance: 1.5–2%). The head of the pup was fixed into a stereotaxic apparatus using two plastic bars mounted on the nasal and occipital bones with dental cement. The bone over the PFC (0.8 mm anterior to bregma, 0.1–0.5 mm right to the midline) and the CA1 area of the i/vHP (0.8–1.0 mm anterior to λ, 3.5–3.8 mm right to the midline) was carefully removed by drilling holes of 0.5-mm diameter. Four-shank optoelectrodes with 4 × 4 recording sites (0.4–0.8 MΩ impedance, 0.1-mm spacing, 0.125-mm intershank spacing; NeuroNexus), aligned with optical fibers (50 µm in diameter) and ending 200 µm above the top recording sites, were inserted into PL at a depth of 2.0 mm from the skull surface. One silver wire was inserted into cerebellum to serve as ground and reference electrode. Before signal acquisition, a recovery period of 15 min after electrode insertion was provided. In PL, the two medial shanks were located into layer 2/3, whereas the lateral shanks were located into layer 5/6. Extracellular signals were bandpass filtered (0.1 Hz to 5 kHz) and digitized (32 kHz) with a multichannel extracellular amplifier (Digital Lynx SX, Neuralynx) and the Cheetah acquisition software (Neuralynx).

Pulsatile (laser on-off, 5 ms, 8 Hz, 3 s) or ramp (linearly increasing power, 3 s) light stimulations *in vivo* were performed with an Arduino uno controlled laser system (473-nm wavelength, Omicron), which was coupled with a 50 µm (four-shank electrodes) diameter light fiber (Thorlabs). Each type of stimulation was repeated 60 times with an interval of 7 s. Laser power was measured and adjusted to the range of 0.75–2.5 mW at the fiber tip.

### Electrophysiological recordings and optogenetic manipulation *in vitro*

For patch-clamp recordings, pups were anaesthetized with 5% isoflurane and decapitated. Brains were rapidly removed and placed in ice-cooled oxygenated (95% O_2_/5% CO_2_) high-sucrose-based artificial CSF (ACSF) containing the following: 228 mm sucrose, 2.5 mm KCl, 1 mm NaH_2_PO_4_, 26.2 mm NaHCO_3_, 11 mm glucose, 0.5 mM CaCl_2_, and 7 mm MgSO_4_ (310 mosmol/kg H_2_O). Coronal brain slices (300 μm) were prepared using a vibratome (Leica VT 1000S). Slices were allowed to recover in oxygenated ACSF containing the following: 119 mm NaCl, 2.5 mm KCl, 1 mm NaH_2_PO_4_, 26.2 mm NaHCO_3_, 11 mm glucose, 2.5 mM CaCl_2_, and 1.3 mm MgSO_4_ (310 mOsmol/kg H_2_O) at 33°C for at least 30 min, then kept at RT (∼22°C) for at least another 60 min before recordings. Slices were transferred to the recording chamber and continuously perfused with oxygenated standard ACSF (2–3 ml/min) at RT.

Whole-cell recordings were made from neurons located in PL and HP. The location and neuronal morphology served to identify the PL layers. PL-projecting neurons in CA1 of i/vHP were identified by CTB555 labeling after tracer injection into PL. Two to three coronal slices were used per animal and chosen according to the coordinates relative to Bregma (for PL, AP: +1.70 to +0.7 mm; for HP, −3.0 to −4.0 mm). Slices were visualized using an upright microscope (BX50WI, Olympus Optical) and with infrared and differential interference contrast optics. All recordings were performed from pyramidal neurons identified according to their shape, spiking pattern, and action potential (AP) width. Borosilicate glass patch pipettes (4–8 MΩ) were filled with K-gluconate-based solution containing the following: 130 mm K-gluconate, 10 mm HEPES, 0.5 mm EGTA, 4 mm Mg-ATP, 0.3 mm Na-GTP, and 8 mm NaCl (285 mosmol/kg H_2_O, pH adjusted to 7.4 with KOH) and 0.3−0.5% biocytin for *post hoc* morphologic identification of recorded cells. Recordings were performed with an EPC 10 amplifier and PatchMaster software v2x73.1 (HEKA Elektronik), filtered at 2.9 kHz using a Bessel filter, and sampled at 10 kHz. All potentials were corrected for the liquid junction potential of the gluconate-based electrode solution, which, according to our measurement, was −8.65 mV. The resting membrane potential (RMP) was measured immediately after obtaining the whole-cell configuration. Unless otherwise noted, all experiments were conducted at a membrane potential of −70 mV under voltage clamp conditions. To measure the basic properties of the membrane, 600-ms-long hyperpolarizing or depolarizing current pulses ranging from −100 to 120 pA in a 20-pA step were applied. Access resistance (R_s_) was monitored under voltage-clamp conditions by analyzing capacitive transients during 5-ms-long square wave depolarizing pulses. Recordings were included only when a GΩ seal formed before whole-cell access with R_s_ of < 30 MΩ. Cells with R_s_ changes >25% were excluded from further investigation. Spontaneous EPSC (sEPSC) events were recorded at a holding potential of −70 mV. None of the investigated neurons showed spontaneous firing at RMP.

For optogenetic stimulation *in vitro*, 470-nm light pulses were applied with a CoolLED system (pE-2) attached to the upright microscope. Maximal light output at 470 nm was measured at 2 mW with an optical power meter (Thorlabs). For stimulation of hippocampal afferents targeting PL neurons, light pulses (3-ms, 5-ms 10-ms, 15-s interval) were repetitively applied every 15 s for up to 10 times. For the investigation of short-term synaptic plasticity, train pulses consisted of 2-s-long light pulses at 2, 4, and 8 Hz repeated every 15 s for up to five times. The induced EPSCs and IPSCs were voltage-clamp recorded at −70 and +10 mV, respectively. To block AMPA/kainate receptors, 10 μm 6-cyano-7-nitroquinoxaline-2, 3-dione (CNQX) was added to the recording chamber solution.

### Histology and immunohistochemistry

Briefly, P8–P10 and P20–P24 mice were anesthetized with 10% ketamine/2% xylazine in 0.9% NaCl solution (10 µg/g body weight, i.p.) transcardially perfused with Histofix containing 4% PFA. Brains were postfixed with 4% PFA for 24 h and sectioned coronally at 100 µm for reconstruction of the position of electrodes, or 50 µm for further staining. Sections for staining were collected in three equally spaced series. To reduce the redundancy of information of neighbor slices, only one of the series was mounted or used for subsequent staining and analysis.

For immunohistochemistry, free floating slices were permeabilized and blocked with PBS containing 0.3% Triton X-100 (Sigma-Aldrich), 5% normal bovine serum (Jackson ImmunoResearch). Subsequently, slices were incubated overnight with mouse monoclonal Alexa Fluor 488-conjugated antibody against NeuN (1:100, MAB377X; Merck Millipore), Alexa Fluor 488-conjugated streptavidin (1:1000, Merck Millipore), or rabbit polyclonal primary antibody against GABA (1:1000, A2052; Sigma-Aldrich), polyclonal guinea-pig antibody against VGLUT1 (1:1000, Synaptic Systems), rabbit polyclonal primary antibody against lectin (1:1000, A2052; Sigma-Aldrich) followed by 2-h incubation with Alexa Fluor 568 goat anti-rabbit IgG secondary antibody (1:500, A11008; Merck Millipore), Alexa Fluor 568 goat anti-guinea pig (1:500, Invitrogen), Alexa Fluor 568 donkey anti-rabbit (1:500, Life Technologies) and Alexa Fluor 568-conjugated streptavidin (1:1000, Merck Millipore). DAPI (1:500) was added to the second antibody for the nuclear labeling. Finally, slices were transferred to glass slides and covered with Vecta-Shield (Vector Laboratories). To avoid cross-reactivity between the anti-lectin primary antibody and other antibodies, sections were firstly incubated with anti-lectin and then underwent subsequent biotinylation and streptavidin treatment steps. Following the last wash, sections were again blocked for 2 h.

For BDA staining, sections (prepared as described above) were rinsed in PBS (0.125 m, pH 7.4–7.6) for 10 min, treated with peroxide solution (3% peroxide, 10% methanol in 0.125 m PB) for 10 min to quench any endogenous peroxidases within the tissue, and rinsed again in PB three times for 10 min each. Subsequently, the sections were washed in PBS containing 0.5% Triton X-100 and incubated with avidin biotinylated enzyme complex (Vectastain ABC kit; Vector) at RT (90 min) or overnight at 4°C according to the manufacturer's instructions. After rinsing in Tris-HCl (pH 7.4), the sections were further incubated with DAB working buffer (DAB peroxidase substrate kit, Vector Laboratories) at RT for 2–10 min. After the signal was detected, all sections were rinsed with Tris-HCl, mounted on slides, dehydrated, cleared in xylenes, coverslipped, and viewed with brightfield microscopy. In some cases, nuclear staining was necessary to aid the delineation of brain regions.

### Imaging

Wide-field fluorescence was performed to reconstruct the position of recording electrode in brain slices of investigated pups. For DAB staining, all bright field images were obtained using a Zeiss imager M1 microscope (Zeiss) with identical settings. Bright field photomicrographs were imported into FIJI and their contrast and brightness were adjusted. Axons were manually traced using FIJI. Area and layer borders were set by superimposing photomicrographs of BDA sections with another series of sections that processed for nuclear staining. HP was stained with streptavidin and DAPI, injection sites in HP were examined and the number of injected neurons was counted. The number of stained neurons was averaged over three hippocampal slices (every third 50-µm-thick slices from the series containing the HP). The density of hippocampal axons (µm/mm^2^) in the PL was normalized to the number of stained neurons in the CA1 and the values were given as µm/mm^2^/cell. In the retrograde tracing experiment, all FG-positive cells in the CA1 were quantified automatically using custom-written algorithms in FIJI, and confirmed by visual inspection. Subsequent analyses of tracing were performed at animal level.

### Data analysis

Electrophysiological data were imported and analyzed offline using custom-written tools in MATLAB software version 7.7 (MathWorks). Data were bandpass filtered [500–5000 Hz for spike analysis or 1–100 Hz for local field potentials (LFPs)] using a third-order Butterworth filter forward and backward to preserve phase information before down-sampling to 1000 Hz to analyze LFP.

#### Power spectral density

For power spectral density analysis, 1-s-long windows of network oscillations were concatenated and the power was calculated using Welch's method with non-overlapping windows. For optical ramp stimulation, we compared the average power during the 1.5-s-long time window preceding the stimulation to the last 1.5-s-long time window of light-evoked activity.

#### Single unit activity (SUA)

SUA was detected and clustered using klusta (Rossant et al., 2016) and manually curated using phy (https://github.com/cortex-lab/phy). Data were imported and analyzed using custom-written tools (https://github.com/OpatzLab/HanganuOpatzToolbox) in MATLAB.

#### Firing rate

The firing rate was computed by dividing the total number of spikes by the duration of the analyzed time window. For optical pulsatile stimulations, modulation index (MI) of firing rate was calculated as (Firing_during-stimulation_ – Firing_pre-stimulation_)/(Firing_during-stimulation_ + Firing_pre-stimulation_).

#### Membrane properties

Analysis of data resulted from patch-clamp recordings was performed offline using custom-written scripts in MATLAB. For all recorded neurons, RMP, input resistance (R_in_), membrane time constant (τ_m_), membrane capacity (C_m_), R_s_, AP amplitude, halfwidth, and firing threshold were calculated. R_in_ was calculated according to Ohm's law by dividing the resulting potential changes by the amplitude of the applied current (−60 pA). τ_m_ was calculated by fitting a monoexponential function to the induced potential deflection. C_m_ was calculated by dividing τ_m_ by R_in_. Firing threshold voltage was considered at the point where the depolarization speed firstly exceeded 10 mV/ms. AP amplitude was measured from threshold to peak, with the half-width measured at half this distance. Firing rate was calculated during a 600-ms-long depolarization of the cells by 80-pA current injection. Sag amplitude was calculated for each cell as the proportional difference between the initial voltage response (i.e., during the first 200 ms of the current pulse) and the steady state response (averaged for 100 ms) to a hyperpolarizing current pulse of −100 pA. Data from PL-projecting neurons and randomly selected neurons in CA1 of i/vHP were pooled together, since no significant differences in the passive and active membrane properties were detected between the two groups.

#### Synaptic activity

Synaptic events were automatically detected automatically on template parameters (Pernía-Andrade et al., 2012) and manually examined to exclude false positive events. Events were excluded if the amplitude was < 3 pA. Interevent interval (event frequency) and event amplitude were analyzed and compared between groups. Light-evoked EPSCs (eEPSCs) were averaged over 10–20 stimuli. Their peak amplitude and onset (i.e., delay between light stimulus and time point at which the response speed exceeded 10 pA/ms) were calculated. The coefficient of variation (CV) for a given measured variable was defined as the ratio between the standard deviation and the average value of 10–20 individual responses to light stimulation.

#### Statistics

Statistical analyses were performed in MATLAB environment. Data were tested for significant differences using one-way repeated-measures ANOVA followed with Bonferroni-corrected *post hoc* analysis. Data with non-normal distribution (only the eEPSC amplitude) were tested with the nonparametric ANOVA followed by Bonferroni-corrected *post hoc* analysis. The effect of experimental groups and layers on the properties of sEPSC was tested using two-way ANOVA followed by Bonferroni-corrected *post hoc* analysis. Values were considered outliers and removed when their distance from the 25th or 75th percentile exceeded 1.5 times the interquartile interval. Data are presented as mean ± SEM. Significance levels of *p* < 0.05 (*), *p* < 0.01 (**), or *p* < 0.001 (***) were used.

## Results

### Anatomical characterization of hippocampal projections targeting the PL cortex in control and dual-hit GE mice

To gain insight into prefrontal-hippocampal communication during development in dual-hit GE mice, we first performed an in-depth structural analysis of axonal projections that link the two regions. In adult mice, the PL subdivision of medial PFC and HP interacts along multiple multi-synaptic routes. The main route of communication is the dense ipsilateral monosynaptic projection from HP to PL, lacking a feedback equivalent (Jay and Witter, 1991; Cenquizca and Swanson, 2007). However, the developmental profile of these unidirectional projections is still poorly understood.

**Figure 1.**
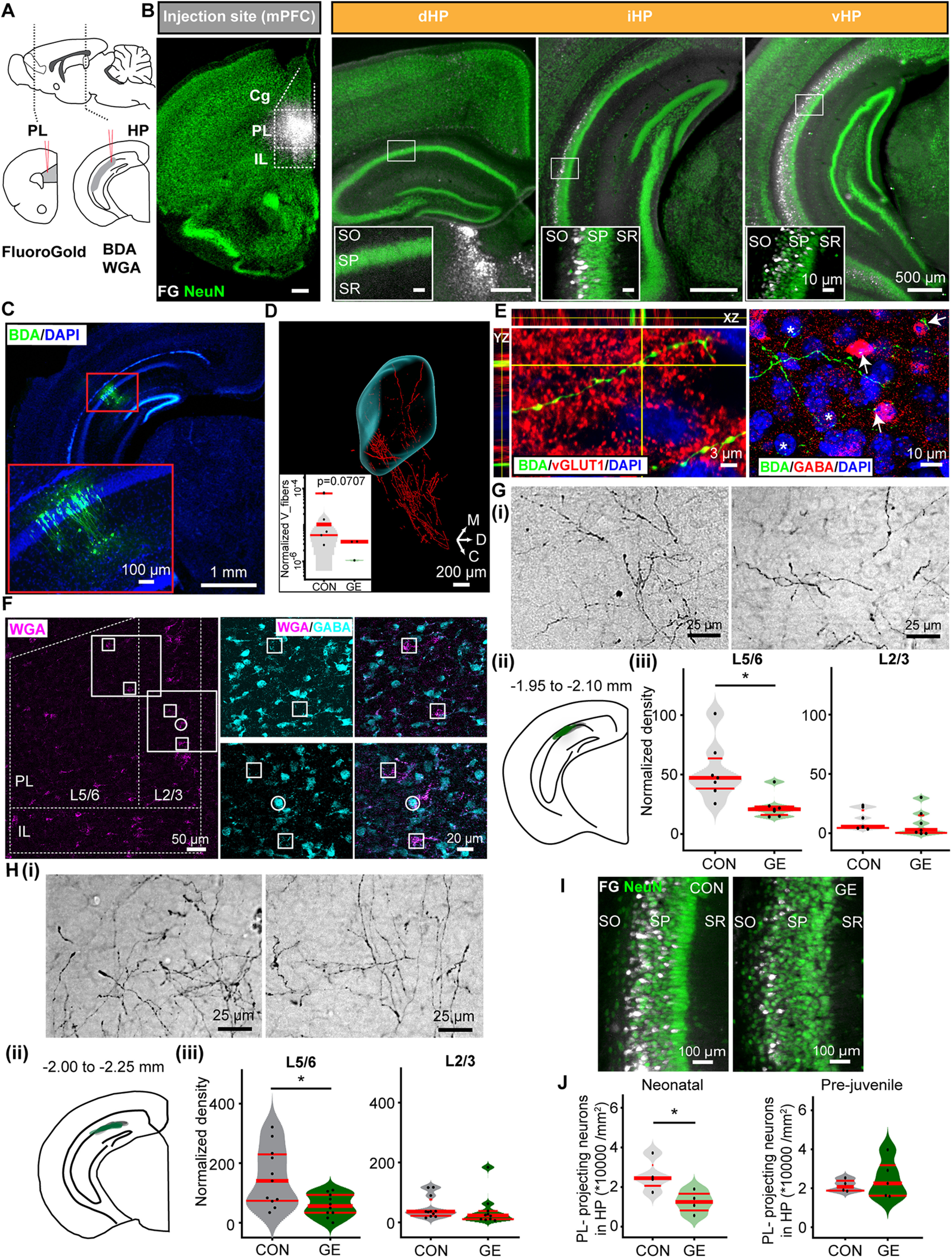
Organization and developmental dynamics of hippocampal innervation of PL in neonatal and prejuvenile dual-hit GE mice. ***A***, Schematic representation of tracing protocols. Mice were injected unilaterally with the retrograde tracer FG into PL or with either the anterograde tracer BDA or the transsynaptic tracer WGA into HP. ***B***, left, Fluorescent images of FG (white) injection site in the PL of 50-µm-thick coronal slice from a P10 mouse when costained with NeuN (green). Right, Corresponding NeuN-stained coronal slices (50-µm-thick) including the dHP, iHP, and vHP with retrogradely stained cells (white). Scale bar: 500 μm. Insets, FG-stained neurons (white) shown at higher magnification. Scale bar: 100 μm. ***C***, Photograph of a representative BDA injection into the i/vHP of a P10 mouse visualized by streptavidin staining (green) and costained with DAPI (blue) in a 50-µm-thick coronal slice. ***D***, 3D reconstruction of hippocampal axons (tDimer, red) into the PL (cyan surface) in a cleared P10 mouse brain. PL volume was delimited according to nuclei staining (DRAQ5; data not shown). Inset, logarithmic violin plots depicting the relative space occupancy of hippocampal fibers within PL, normalized to the number of transfected neurons in i/vHP. ***E***, left, Orthogonal views of the Z-stack (YZ, XZ) images illustrating BDA-positive boutons (green) that colocalized with vGLUT1 (red). Right, Confocal images displaying BDA-positive boutons (green) on GABA-positive (red) somata (arrows) and GABA-negative neurons (asterisk). ***F***, left, Representative example of WGA staining (magenta) in a 50-µm-thick coronal slice including the PFC of a P10 mouse that transsynaptically labeled neurons targeted by hippocampal axons. Dotted lines mark the borders of the two subdivisions as well the PL layers. Right, Photographs displaying the colocalization of WGA (magenta) and GABA (cyan) staining for GABA-positive neurons (circle). GABA-negative but WGA-positive neurons are marked by squares. ***Gi***, Photograph of BDA-labeled hippocampal terminals (black) targeting deep layers of PL from a P10 CON (left) and a P10 GE (right) mouse, respectively. ***ii***, Schematic illustrating the extent of the BDA injections into the i/vHP of neonatal CON (gray) and dual-hit GE (green) mice. ***iii***, Violin plots of the normalized density of hippocampal terminals (in µm/mm^2^/cell) in layer 2/3 and layer 5/6 of PL averaged for all investigated neonatal CON and GE mice. ***H***, Same as ***G***, for prejuvenile mice. ***I***, Photograph of FG-labeled neurons (white) in the CA1 area of i/vHP in a 50-µm-thick NeuN-stained (green) coronal slice from a P10 CON and a P10 GE mouse, respectively. ***J***, Violin plots of the normalized density of PL-projecting neurons in the hippocampal CA1 area averaged for all investigated neonatal (left) and prejuvenile (right) CON and GE mice. PL: prelimbic cortex, IL: infralimbic cortex, Cg: cingulate cortex, SO: stratum oriens, SP: stratum pyramidale, SR: stratum radiatum. Single data points are represented as dots and the red horizontal bars in violin plots correspond to the median and the 25th and 75th percentiles; *p < 0.05.

To close this knowledge gap, we performed retrograde and anterograde staining of the hippocampal projections accompanied by path tracking during early neonatal development (Fig. 1*A*). First, we used the retrograde tracer FG that was iontophoretically injected into the PL of control mice (CON; *n* = 4) at P7. Three days later, we detected labeled neurons in PL (Fig. 1*B*), with minimal diffusion to neighboring areas, such as infralimbic cortex (IL). FG injection labeled few cells in dorsal HP (dHP), but labeled cell density augmented along the fronto-caudal axis and peaked at the level of i/vHP, which is consistent with our previous results (Ahlbeck et al., 2018). FG-positive neurons were not uniformly distributed over hippocampal areas but mainly concentrated in the deep layers of CA1 region, close to stratum oriens (SO; Fig. 1*B*). Second, we iontophoretically injected the anterograde tracer BDA into the hippocampal CA1 region of P7 CON mice (*n* = 7; Fig. 1*C*). Three days later, BDA-labeled axonal terminals were detected in PL, accumulating in, yet not exclusively restricted to layer 5/6. The path and distribution of hippocampal axonal streamlines terminating in PL were revealed in whole-brain imaging after electrophoretic tissue clearing and confocal fluorescence microscopy (Fig. 1*D*; Multimedia 1). These results show that already at neonatal age, the distribution of axonal projections over PL layers resembles connectivity previously described for adult mice (Parent et al., 2010; Padilla-Coreano et al., 2016; Liu and Carter, 2018). The BDA-positive terminals had large boutons (Fig. 1*E*) and were vGLUT1 immunopositive (Fig. 1*E*), indicating that hippocampal projections targeting PL are glutamatergic. They seem to target both interneurons and pyramidal neurons, as shown by close proximity of BDA-stained axons to both GABA-positive and -negative cells (Fig. 1*E*). To confirm these results and identify first-order postsynaptic neurons in PL innervated by hippocampal axons, we injected the transsynaptic marker WGA into the HP of CON mice (*n* = 3). WGA-positive postsynaptic neurons have been identified in both layer 5/6 and layer 2/3 of PL (Fig. 1*F*). GABA costaining showed that the large majority (97.9%, 423 out of 432) of WGA-positive neurons were GABA-negative and very few targeted neurons (2.1%, 9 out of 432) were GABA-positive (Fig. 1*F*). These results indicate that, already at the end of the first postnatal week, hippocampal neurons located in SO of CA1 area project to PL, where they mainly, but not exclusively, target pyramidal neurons.

Movie 1. Hippocampal projections to the PL cortex. 3D reconstruction of traced hippocampal terminals in PL from a neonatal CON mouse after tissue clearing. tDimer expression (red), nuclei staining (cyan). The PL (cyan surface) was outlined according to nuclei staining. tDimer-labeled fibers were segmented and the signal was enhanced. 10.1523/JNEUROSCI.2157-20.2021.video.1

In dual-hit GE mice (*n* = 7), the overall pattern of hippocampal innervation in PL was similar to that identified in neonatal CON mice. However, when monitoring the density of axonal projections from i/vHP to PL major differences were detected between the two groups. In cleared brains, fewer projections were visualized and quantified in the PL of neonatal GE mice when compared with CON mice (Fig. 1*D*, inset). This was confirmed after BDA staining. Fewer projections have been detected in the deeper PL layers of neonatal GE mice (*n* = 7 mice) than CON mice (*n* = 7 mice; in µm/mm^2^/cell, CON: 53.07 ± 9.41; GE: 22.67 ± 3.76; *F*_(1,12)_ = 9.00, *p* = 0.011; Fig. 1*G*). In contrast, the density of hippocampal projections in the upper layers of PL was similar (in µm/mm^2^/cell, CON: 10.79 ± 3.17; GE: 8.57 ± 4.31; *F*_(1,12)_ = 0.17, *p* = 0.686) in all investigated mice, yet these layers are not the major target of CA1 innervation and therefore, the overall density here was low (Fig. 1*G*). Thus, sparser connectivity from HP to PL is present in neonatal GE mice.

These connectivity deficits persisted during the entire development, as shown by the results of similar BDA injections in the i/vHP of P21 prejuvenile GE and CON mice (Fig. 1*H*). At P24, the density of hippocampal projections, especially in deeper PL layers, strongly increased in all mice, yet it was still smaller (in µm/mm^2^/cell, CON layer 5/6: 153.27 ± 30.11; GE layer 5/6: 58.26 ± 12.03; *F*_(1,19)_ = 7.969, *p* = 0.0108; Fig. 1*H*) in GE mice (*n* = 10 mice) when compared with CON mice (*n* = 11 mice). In contrast, no differences between the groups were detected for the rather sparse innervation of PL layer 2/3 (in µm/mm^2^/cell, CON: 50.08 ± 11.57; GE: 39.41 ± 16.93; *F*_(1,19)_ = 0.280, *p* = 0.603).

To answer the question of whether the sparser hippocampal innervation of layer 5/6 in GE mice relates to fewer projecting neurons in CA1 area or cropped arborization of projections, we injected the retrograde tracer FG into PL (CON: *n* = 4 mice, GE: *n* = 4 mice) and quantified the density of stained hippocampal neurons (Fig. 1*I*,*J*). The density of PL-projecting neurons was significantly lower in neonatal GE mice when compared with CON (in *10,000/mm^2^, CON: 2.59 ± 0.414; GE: 1.24 ± 0.28; *F*_(1,6)_ = 7.20, *p* = 0.0364). In contrast, the density of retrogradely stained CA1 neurons was similar in CON (*n* = 4 mice) and GE (*n* = 5 mice) mice at prejuvenile age (in *10,000/mm^2^, CON: 2.13 ± 0.16; GE: 2.47 ± 0.45, *F*_(1,7)_ = 0.409, *p* = 0.543). A similar density of PFC-projecting neurons but a lower density of hippocampal projections in PL suggests that the projections are less arborized in prejuvenile GE mice.

These data show that the hippocampal innervation of PL is impaired in GE mice, with fewer CA1 neurons projecting to PL at neonatal age and less arborized projections toward the end of prejuvenile development.

### Region-dependent and age-dependent cellular dysfunction within hippocampal-PL circuits of dual-hit GE mice

The structural deficits observed in dual-hit GE mice along development lead to the question of whether early neuronal function is affected as well. Abnormal cellular activity might underlie the decreased functional communication between PL and HP that has been previously reported in these mice at neonatal age (Hartung et al., 2016; Xu et al., 2019).

**Figure 2.**
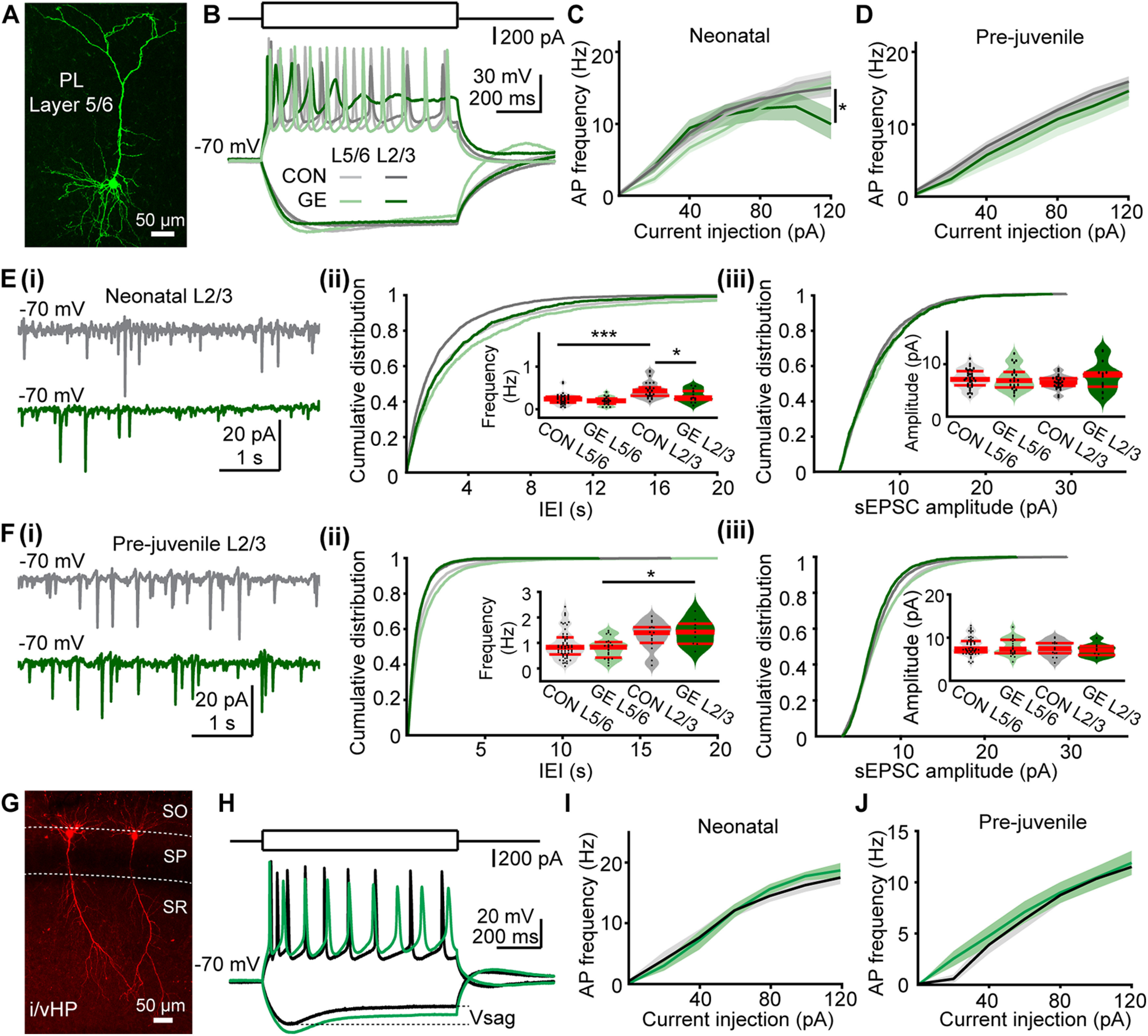
Passive and active membrane properties as well as synaptic inputs of PL and hippocampal neurons from neonatal and prejuvenile CON and GE mice *in vitro*. ***A***, Confocal image showing a biocytin-filled pyramidal neuron in layer 5/6 of PL from a P10 CON mouse. ***B***, Representative voltage responses to the injection of hyper- and depolarizing current pulses (holding membrane potential of −70 mV) of pyramidal neurons in layer 5/6 (light gray), layer 2/3 (dark gray) of the PL from P10 CON mice as well as for pyramidal neurons in layer 5/6 (light green), layer 2/3 (dark green) of the PL from P10 GE mice. ***C***, Firing rate in relationship to current injection displayed for layer 5/6 (*n* = 33) and layer 2/3 (*n* = 25) neurons from neonatal CON mice as well as for layer 5/6 (*n* = 20) and layer 2/3 (*n* = 14) from neonatal GE mice; * indicates the comparison of firing rate of layer 2/3 neurons in response to 120-pA current injection between CON and GE. ***D***, Same as ***C***, for prejuvenile mice (*n* = 28 for CON layer 5/6, *n* = 15 for CON layer 2/3, *n* = 20 for GE layer 5/6, *n* = 12 for GE layer 2/3). ***Ei***, Representative traces of sEPSCs recorded from layer 2/3 pyramidal neurons from P10 CON (gray) and GE (green) mice. ***ii***, Cumulative probability distribution of interevent intervals (IEIs) and violin plots (inset) of sEPSCs frequencies averaged for all prefrontal neurons in CON and GE mice. ***iii***, Same as ***ii*** for sEPSC amplitude. ***F***, Same as ***E***, for prejuvenile mice. ***G***, Confocal image showing a biocytin-filled pyramidal neuron in the CA1 area of i/vHP from a P10 CON mouse. SO: stratum oriens, SP: stratum pyramidale, SR: stratum radiatum. ***H***, Representative voltage responses to the injection of hyper- and depolarizing current pulses (holding membrane potential of −70 mV) of CA1 pyramidal neurons from P10 CON (black) and GE (green) mice. ***I***, Firing rate in relationship to current injection displayed for CA1 neurons from neonatal CON (*n* = 15, black) and GE (*n* = 10, green) mice. ***J***, Same as ***I***, for prejuvenile CA1 neurons (*n* = 12 for CON, *n* = 9 for GE). Single data points are represented as dots and the red horizontal bars in violin plots correspond to the median and the 25th and 75th percentiles; *p < 0.05, **p < 0.01, ***p < 0.001.

To test this hypothesis, we first monitored the membrane properties of PL and hippocampal CA1 neurons in neonatal (P8–P10) and prejuvenile (P20–P24) CON and GE mice. For this, we performed whole-cell patch-clamp recordings from visually identified and biocytin-stained neurons in coronal slices including PL or i/vHP. In the PL, cells in the upper layers (i.e., layers 2 and 3) as well as deeper layers (i.e., layers 5 and 6) have been recorded. The pyramidal shape and the orientation of dendrites monitored postmortem after biocytin staining served as criteria to unequivocally classify the investigated cells as pyramidal neurons (Fig. 2*A*,*G*). Already at neonatal age, the passive and active membrane properties of PL pyramidal neurons differed between neonatal CON and GE mice (CON layer 5/6: *n* = 33 cells, GE layer 5/6: *n* = 20 cells, CON layer 2/3: *n* = 27 cells, GE layer 2/3: *n* = 14 cells). The RMP of upper layer neurons was more depolarized, the AP amplitude smaller, and the AP halfwidth longer in GE when compared with CON mice (Table 1). All neurons fired overshooting APs in response to sustained depolarization by intracellular current injection (Fig. 2*B*). No difference in firing rate in response to depolarizing current injection was detected between CON and GE (Fig. 2*C*). With ongoing maturation, the cellular properties of PL neurons evolved in all mice (CON layer 5/6: *n* = 57 cells, GE layer 5/6: *n* = 23 cells, CON layer 2/3: *n* = 15 cells, GE layer 2/3: *n* = 9 cells; Fig. 2*D*). The differences between CON and GE mice diminished with age, solely the RMP of PL layer 2/3 pyramidal neurons being more hyperpolarized in prejuvenile GE mice when compared with CON (Table 1).

**Table 1.** Passive and active membrane properties of prefrontal and hippocampal neurons from neonatal and prejuvenile CON and GE mice *in vitro*

Membrane Properties			Layer 5/6			Layer 2/3			HP		
			CON	GE	*p*	CON	GE	*p*	CON	GE	*p*
Neonatal (P8–P10)	Passive	R_in_ (MΩ)	401.8 ± 19.5	427.6 ± 35.1	0.5352	471.3 ± 18	536.1 ± 35.8	0.0699	453.8 ± 19.8	361.3 ± 16.1	**0.0008**
		C_m_ (pF)	125.1 ± 5.6	125.6 ± 13.9	0.9693	94.2 ± 4.4	90.5 ± 3.3	0.5804	118.1 ± 5.8	109.7 ± 5.52	0.2559
		τ_m_ (ms)	83.9 ± 4.3	81.4 ± 8.0	0.7876	67.2 ± 3.5	73.4 ± 4.6	0.2879	49.6 ± 2.6	41.4 ± 2.9	**0.0376**
		RMP (mV)	−68.4 ± 0.7	−67.2 ± 0.9	0.3986	−69.8 ± 0.9	−65.8 ± 1.1	**0.0096**	−69.2 ± 0.8	−69.0 ± 0.7	0.3016
		Sag (%)	10.2 ± 0.9	12.8 ± 2.5	0.2225	4.4 ± 0.62	4.58 ± 0.38	0.8457	23.7 ± 1.2	29.3 ± 1.2	**0.0028**
	Active	AP threshold (mV)	−42.4 ± 1.3	−42.8 ± 2.1	0.9080	−38.8 ± 1.2	−39.1 ± 1.3	0.8544	−40.5 ± 1.0	−43.0 ± 1.3	0.1148
		AP amplitude (mV)	71.5 ± 1.3	69.4 ± 2.2	0.8525	66.8 ± 1.4	59.5 ± 1.8	**0.0028**	80.7 ± 1.4	81.9 ± 1.4	0.5591
		AP halfwidth (ms)	3.56 ± 0.15	3.40 ± 0.18	0.5970	2.85 ± 0.06	3.38 ± 0.15	**0.00062**	2.05 ± 0.04	2.25 ± 0.07	**0.0085**
		Rheobase (pA)	43.5 ± 3.6	40.4 ± 45.0	0.6716	43.1 ± 2.9	46.6 ± 6.8	0.5710	58.3 ± 2.7	59.3 ± 2.6	0.7863
		Firing rate (Hz)	12.3 ± 0.7	11.3 ± 1.1	0.4956	12.5 ± 1.0	11.3 ± 1.6	0.4945	12.5 ± 0.7	12.3 ± 0.8	0.7438
Prejuvenile (P20–P24)	Passive	R_in_ (MΩ)	222.2 ± 11.5	188.9 ± 17.9	0.1146	235.4 ± 13.2	237.8 ± 9.7	0.8938	239.0 ± 19.6	211.9 ± 24.7	0.3703
		C_m_ (pF)	178.2 ± 12.5	184.7 ± 13.9	0.7547	131.3 ± 9.37	115.2 ± 8.4	0.2409	174.9 ± 24.0	131.9 ± 22.8	0.1997
		τ_m_ (ms)	59.6 ± 5.8	45.9 ± 4.7	0.1353	46.9 ± 3.64	44.5 ± 2.9	0.6337	37.7 ± 1.6	33.2 ± 5.1	0.3286
		RMP (mV)	−69.5 ± 0.5	−69.3 ± 0.5	0.8084	−66.9 ± 0.5	−69.7 ± 0.4	**0.00076**	−64.9 ± 1.1	−63.5 ± 2.0	0.5000
		Sag (%)	16.2 ± 1.7	18.4 ± 2.7	0.4839	6.4 ± 1.6	4.3 ± 0.7	0.3337	27.3 ± 2.0	21.1 ± 2.8	0.0664
	Active	AP threshold (mV)	−46.1 ± 1.3	−48.4 ± 2.0	0.3297	−44.5 ± 1.2	−46.4 ± 1.7	0.3370	−45.4 ± 1.8	−49.3 ± 1.9	0.1325
		AP amplitude (mV)	90.1 ± 1.1	90.9 ± 1.6	0.6539	88.5 ± 1.1	86.8 ± 1.3	0.3388	97.1 ± 2.7	96.8 ± 4.4	0.9528
		AP halfwidth (ms)	1.67 ± 0.05	1.53 ± 0.07	0.6839	1.79 ± 0.06	1.65 ± 0.07	0.1186	1.73 ± 0.04	1.74 ± 0.06	0.8715
		Rheobase (pA)	73.8 ± 5.2	78.6 ± 9.3	0.6231	85.1 ± 6.2	89.5 ± 7.8	0.6694	50.7 ± 2.7	48.4 ± 5.1	0.6444
		Firing rate (Hz)	11.4 ± 0.8	10.2 ± 1.3	0.4260	11.8 ± 0.7	10.6 ± 1.1	0.3109	15.0 ± 1.2	15.9 ± 1.8	0.7774

Data are shown as mean ± SEM. Significance was assessed using one-way ANOVA test followed by Bonferroni-corrected *post hoc* test, and the listed *p* values correspond to comparisons between CON and GE mice for the neurons in the same region.

Second, we performed voltage-clamp recordings at a holding potential of −70 mV from PL neurons from neonatal CON and GE mice to assess their synaptic inputs (Fig. 2*E*,*F*). sEPSCs with large amplitude and fast kinetics were recorded in PL neurons from both groups. The occurrence (Fig. 2*Eii*) but not amplitude (Fig. 2*Eiii*) of sEPSCs in PL layer 2/3 was significantly smaller in GE mice (Table 2). The synaptic activity in both areas was comparable between groups in prejuvenile CON and GE mice and only a few differences were detected (Fig. 2*F*; Table 2). These data confirm previous investigations that detected prominent dysfunction within local circuits and morphologic change in the upper PL layers in neonatal GE mice, and less prominent changes in prejuvenile GE mice (Chini et al., 2020).

**Table 2.** Properties of sEPSCs recorded from prefrontal neurons in CON and GE mice *in vitro*

sEPSC properties		L5/6		L2/3		*F* values CON vs GE	*F* values L5/6 vs L2/3	*F* values interaction
		CON	GE	CON	GE			
Neonatal (P8–P10)	Frequency (Hz)	0.24 ± 0.023 ###*p* < 0.0001	0.19 ± 0.022	0.45 ± 0.037 *p = 0.040	0.31 ± 0.041	*F*_(1,92)_ = 8.141 *p* = 0.005	*F*_(1,92)_ = 26.43 *p* < 0.0001	*F*_(1,92)_ = 1.844 *p* = 0.178
	Amplitude (pA)	7.40 ± 0.22	7.45 ± 0.36	6.92 ± 0.36	7.75 ± 0.56	*F*_(1,92)_ = 2.08 *p* = 0.153	*F*_(1,92)_ = 0.08 *p* = 0.773	*F*_(1,92)_ = 1.645 *p* = 0.203
Prejuvenile (P20–P24)	Frequency (Hz)	0.93 ± 0.074	0.79 ± 0.083 *p = 0.018	1.28 ± 0.15	1.41 ± 0.18	*F*_(1,101)_ = 0.002 *p* = 0.967	*F*_(1,101)_ = 14.44 *p* = 0.0002	*F*_(1,101)_ = 1.200 *p* = 0.276
	Amplitude (pA)	7.97 ± 0.27	7.88 ± 0.42	7.45 ± 0.47	7.09 ± 0.53	*F*_(1,101)_ = 0.227 *p* = 0.635	*F*_(1,101)_ = 1.896 *p* = 0.172	*F*_(1,101)_ = 0.077 *p* = 0.782

Data are shown as mean ± SEM. Significance was assessed using two-way ANOVA test followed by Bonferroni-corrected *post hoc* test. The listed *p* values (*) correspond to comparisons CON L5/6 versus GE L5/6 and CON L2/3 versus GE L2/3, whereas *p* values (###) correspond to comparisons CON L5/6 versus CON L2/3, GE L5/6 versus GE L2/3.

Similar investigation of pyramidal neurons in CA1 area of i/vHP showed that their passive and active membrane properties differed between neonatal CON (*n* = 34) and GE neurons (*n* = 27). While the RMP and membrane capacitance values were similar among groups, the input resistance was significantly smaller and the time constant shorter for CA1 neurons of GE mice (Table 1), suggesting lower excitability of these neurons when compared with those from CON mice. This difference is supported by the bigger voltage sag recorded on hyperpolarization in hippocampal neurons from GE mice (Fig. 2*H*; Table 1). The voltage sag mirrors activation of hyperpolarization-activated cyclic nucleotide-gated (HCN) channels that are known to control neuronal excitability (Brennan et al., 2016). The firing rate in response to sustained depolarization was similar in CON and GE mice (Fig. 2*I*; Table 1). Solely the AP width was higher in neurons from GE mice. At prejuvenile age, no major differences in membrane properties and firing rate of hippocampal neurons were detected between CON and GE mice (Fig. 2*J*, *n* = 12 for CON, *n* = 9 for GE; Table 1).

These data indicate that a mild cellular dysfunction of prefrontal and hippocampal neurons is present in neonatal GE mice and diminishes with age.

### Weaker efficiency of hippocampal drive to PL cortex in neonatal dual-hit GE mice

To investigate whether, besides structural disruption, the functional connectivity between HP and PL is compromised in GE mice during development, we monitored the responsiveness of PL neurons to the activation of hippocampal terminals.

**Figure 3.**
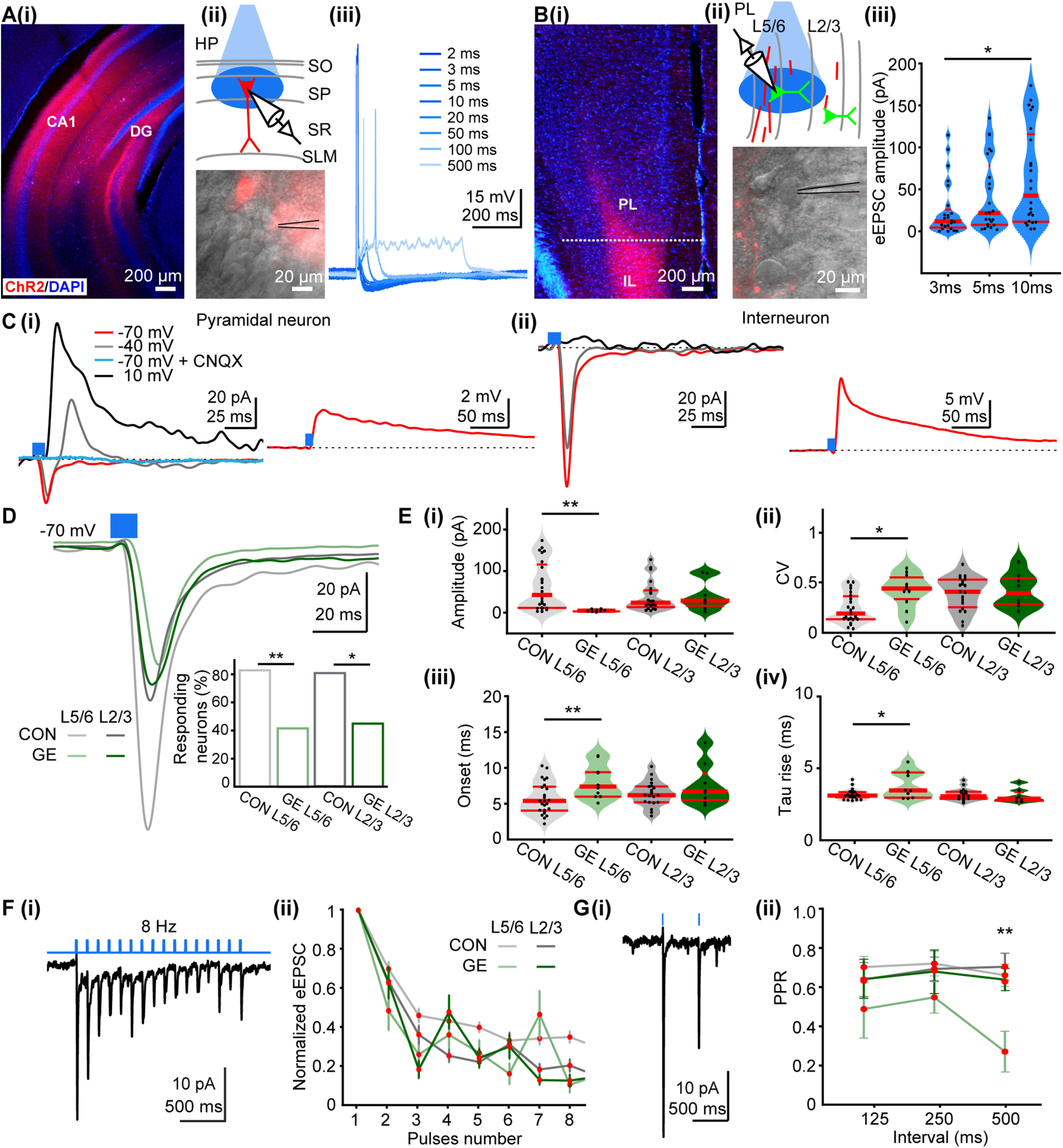
Synaptic properties and plasticity of hippocampal inputs on PL pyramidal neurons in neonatal CON and GE mice. ***Ai***, Representative image showing ChR2 (H134R; red) expression in a DAPI-stained coronal slice from a P10 CON mouse following hippocampal injection at P1. ***ii***, Schematic of light stimulation of hippocampal CA1 neurons expressing ChR2 (H134R; red). ***iii***, Voltage responses of a ChR2-expressing neuron to light stimuli (470 nm, 5–10 mW/mm^2^) of 2–500 ms in duration. ***Bi***, Representative image showing hippocampal axons (red) in PL and IL from a P10 CON mouse following hippocampal injection at P1. ***ii***, Schematic of light stimulation of hippocampal axons in PL. ***iii***, Violin plots of eEPSC amplitudes evoked by light stimuli of 3, 5, 10 ms in duration. Data were collected from layer 5/6 pyramidal neurons (*n* = 24) in PL of CON mice. ***Ci***, left, Representative current responses to light stimulation (blue bar: 10 ms) of HP terminals for a putative pyramidal neuron (red, holding potential of −70 mV; gray, −40 mV, black, 10 mV). The response was abolished by bath CNQX (blue trace). Right, Representative voltage response to light stimulation (blue bar: 10 ms) of HP terminals for a pyramidal neuron. ***ii***, Same as ***Ci*** for a putative interneuron. ***D***, Average eEPSC (holding potential of –70 mV) evoked by light in layer 5/6 (*n* = 24) and layer 2/3 (*n* = 21) neurons from neonatal CON mice as well as for pyramidal neurons in layer 5/6 (*n* = 10) and layer 2/3 (*n* = 8) from neonatal GE mice. Blue bar corresponds to 10-ms light stimulation. Inset, Bar diagram of the percentage of responsive pyramidal neurons in different groups. ***E***, Violin plots showing the (***i***) amplitudes, (***ii***) CV of amplitudes, (***iii***) synaptic delay, and (***iv***) rise tau of eEPSCs averaged for all prefrontal neurons in CON and GE mice. ***Fi***, Representative current response to pulsed light (8 Hz; blue) of a layer 5/6 pyramidal neuron from a P10 CON mouse. ***ii***, Plot of eEPSC amplitude (normalized to the first EPSC amplitude) in response to 8-Hz stimulation averaged for all prefrontal neurons in CON and GE mice. ***Gi***, Representative response to light stimuli (500-ms interstimulus interval) of a layer 5/6 pyramidal neuron from a P10 CON mouse. ***ii***, Plot of PPR at 125-, 250-, 500-ms interstimulus intervals averaged for all prefrontal neurons in CON and GE mice. Single data points are represented as dots and the red horizontal bars in violin plots correspond to the median and the 25th and 75th percentiles; *p < 0.05, **p < 0.01.

In a first step, we focused on cellular processes assessed under *in vitro* conditions. For this, we selectively transfected pyramidal neurons in the HP of P1 CON and GE mice with ChR2 (H134R) and fluorescent protein EYFP (AAV9-hSyn-hChR2(H134R)-EYFP) by micro-injections (Fig. 3*Ai*). Whole-cell patch-clamp recordings from pyramidal CA1 neurons in coronal slices including i/vHP from P8–P10 mice confirmed that blue light (473 nm, 3 ms) pulses reliably evoked APs (Fig. 3*Aii*,*iii*). In line with the results of morphologic investigations, fluorescent axonal terminals of transfected CA1 neurons were detected in the deep and, to a lesser extent, in upper layers of PL (Fig. 3*Bi*). We performed voltage-clamp recordings from visually-identified PL pyramidal neurons and nonpyramidal cells (i.e., putatively interneurons) located in the proximity of terminals during light-stimulation of hippocampal axons (Fig. 3*Bii*). Single pulses of light stimulation evoked prominent EPSCs (eEPSCs) in both pyramidal neurons (Fig. 3*Ci*) and interneurons (Fig. 3*Cii*), the amplitude of which augmented with increasing stimulus duration (3 ms: 24.81 ± 6.70 pA, 5 ms: 42.19 ± 9.02 pA, 10 ms: 63.6 ± 12.12 pA, *F*_(2,69)_ = 8.07, *p* = 0.017, *p* = 0.012, *n* = 24; Fig. 3*Biii*). The eEPSCs recorded from pyramidal neurons had a short latency and fast kinetics and were fully abolished when ionotropic AMPA/kainate receptor antagonists CNQX (10 μm) was added to the extracellular solution (Fig. 3*Ci*). Upon depolarization, the AMPA receptor-mediated events were accompanied by a delayed di-synaptic postsynaptic current.

While light stimulation evoked robust responses in PL neurons of all investigated pups (Fig. 3*D*), detailed analysis examining eEPSCs properties revealed differences between CON and GE mice (Table 3). In both PL layers in GE mice, fewer neurons responded to light stimulation of hippocampal projections (layer 5/6, 41.67%; layer 2/3, 44.44%) when compared with responding neurons in CON (layer 5/6, 82.76%, *p* = 0.0048; layer 2/3, 80.76%, *p* = 0.029). Moreover, the eEPSC evoked in layer 5/6 neurons had not only smaller amplitude in GE versus CON mice (63.6 ± 12.12 vs 9.72 ± 2.41 pA, *p* = 0.0009) but also showed a higher degree of variability on light stimulus as mirrored by the larger CV (Fig. 3*Ei*,*ii*; Table 3). The kinetics of eEPSCs were also disrupted in GE mice, the events having a delayed onset (5.15 ± 0.476 vs 7.99 ± 0.762 ms, *p* = 0.006) and longer rise-time (3.07 ± 0.044 vs 3.60 ± 0.301 ms, *p* = 0.022; Fig. 3*Eiii*,*iv*). In contrast, the properties of light-eEPSCs in layer 2/3 neurons were similar for all investigated mice (Fig. 3*E*; Table 3). The function of hippocampal terminals in PL was further assessed by repetitive stimulation (Fig. 3*Fi*). All PL neurons of neonatal CON and GE neurons responded with a substantial depression of eEPSCs when normalized to the first event (Fig. 3*Fii*). However, the paired-pulse ratio (PPR), a measure of short-term plasticity (STP), for layer 5/6 neurons in GE mice significantly decreased when the stimulation was delivered at a 500-ms interval (0.67 ± 0.057 vs 0.21 ± 0.106, *p* = 0.0083; Fig. 3*G*; Table 3). These results suggest that the hippocampal inputs are less efficient on PL neurons in GE mice.

**Table 3.** Properties of EPSCs evoked by stimulation of hippocampal terminals *in vitro*

Light-evoked EPSCs		CON L5/6	GE L5/6	Neonatal CON L2/3	GE L2/3	*F* values	CON L5/6	GE L5/6	Prejuvenile CON L2/3	GE L2/3	*F* values
Response %		24/29 (82.76%)	10/24 (41.67%)	21/26 (80.76%)	8/18 (44.44%)		23/34 (67.65%)	15/32 (46.88%)	12/21 (57.14%)	6/16 (37.5%)	
Amplitude (pA)		63.6 ± 12.12 **p = 0.001	9.72 ± 2.41	47.9 ± 12.00	40.97 ± 13.6	*F*_(3,59)_ = 14.02 *p* = 0.0029	70.1 ± 13.74 *p = 0.0354	19.7 ± 4.39	43.0 ± 14.32	26.2 ± 15.96	*F*_(3,52)_ = 9.877 *p* = 0.0196
CV		0.236 ± 0.032 *p = 0.016	0.421 ± 0.052	0.390 ± 0.04	0.416 ± 0.066	*F*_(3,59)_ = 5.35 *p* = 0.0026	0.34 ± 0.041 **p = 0.0005	0.60 ± 0.037	0.45 ± 0.063	0.40 ± 0.049	*F*_(3,52)_ = 6.045 *p* = 0.0012
Onset delay (ms)		5.15 ± 0.476 **p = 0.006	7.99 ± 0.762	6.29 ± 0.382	7.55 ± 1.128	*F*_(3,59)_ = 5.00 *p* = 0.0037	4.41 ± 0.508	3.75 ± 0.506 ^##^*p* = 0.0044	4.89 ± 0.378	5.15 ± 0.861	*F*_(3,52)_ = 5.967 *p* = 0.0014
Tau rise (ms)		3.07 ± 0.044 *p = 0.022	3.60 ± 0.301	3.18 ± 0.093	3.06 ± 0.171	*F*_(3,59)_ = 3.20 *p* = 0.030	3.63 ± 0.212	3.20 ± 0.158	3.31 ± 0.183	3.11 ± 0.159	*F*_(3,52)_ = 1.358 *p* = 0.2651
PPR	125 ms	0.70 ± 0.056	0.48 ± 0.15	0.64 ± 0.09	0.63 ± 0.13	*F*_(3,59)_ = 2.28 *p* = 0.5156	1.23 ± 0.09 *p = 0.037	0.94 ± 0.13	0.96 ± 0.12 *p = 0.0086	0.51 ± 0.12	*F*_(3,52)_ = 17.11 *p* = 6.70E-04
	250 ms	0.72 ± 0.06	0.54 ± 0.08	0.69 ± 0.06	0.68 ± 0.15	*F*_(3,59)_ = 3.95 *p* = 0.2672	1.35 ± 0.08	1.19 ± 0.14	1.07 ± 0.15	0.87 ± 0.14	*F*_(3,52)_ = 8.88 *p* = 0.0310
	500 ms	0.66 ± 0.056 **p = 0.0061	0.27 ± 0.104	0.70 ± 0.069	0.62 ± 0.079	*F*_(3,59)_ = 11.09 *p* = 0.0074	1.28 ± 0.111	1.23 ± 0.108	0.98 ± 0.105	1.03 ± 0.174	*F*_(3,52)_ = 5.505 *p* = 0.1384

Data are shown as mean ± SEM. Significance was assessed using one-way ANOVA test followed by Bonferroni-corrected *post hoc* test. The listed *p* values (*p < 0.05, **p < 0.01) correspond to comparisons CON L5/6 versus GE L5/6 and CON L2/3 versus GE L2/3, whereas *p* values (^##^*p* < 0.01) correspond to comparisons CON L5/6 versus CON L2/3, GE L5/6 versus GE L2/3.

**Figure 4.**
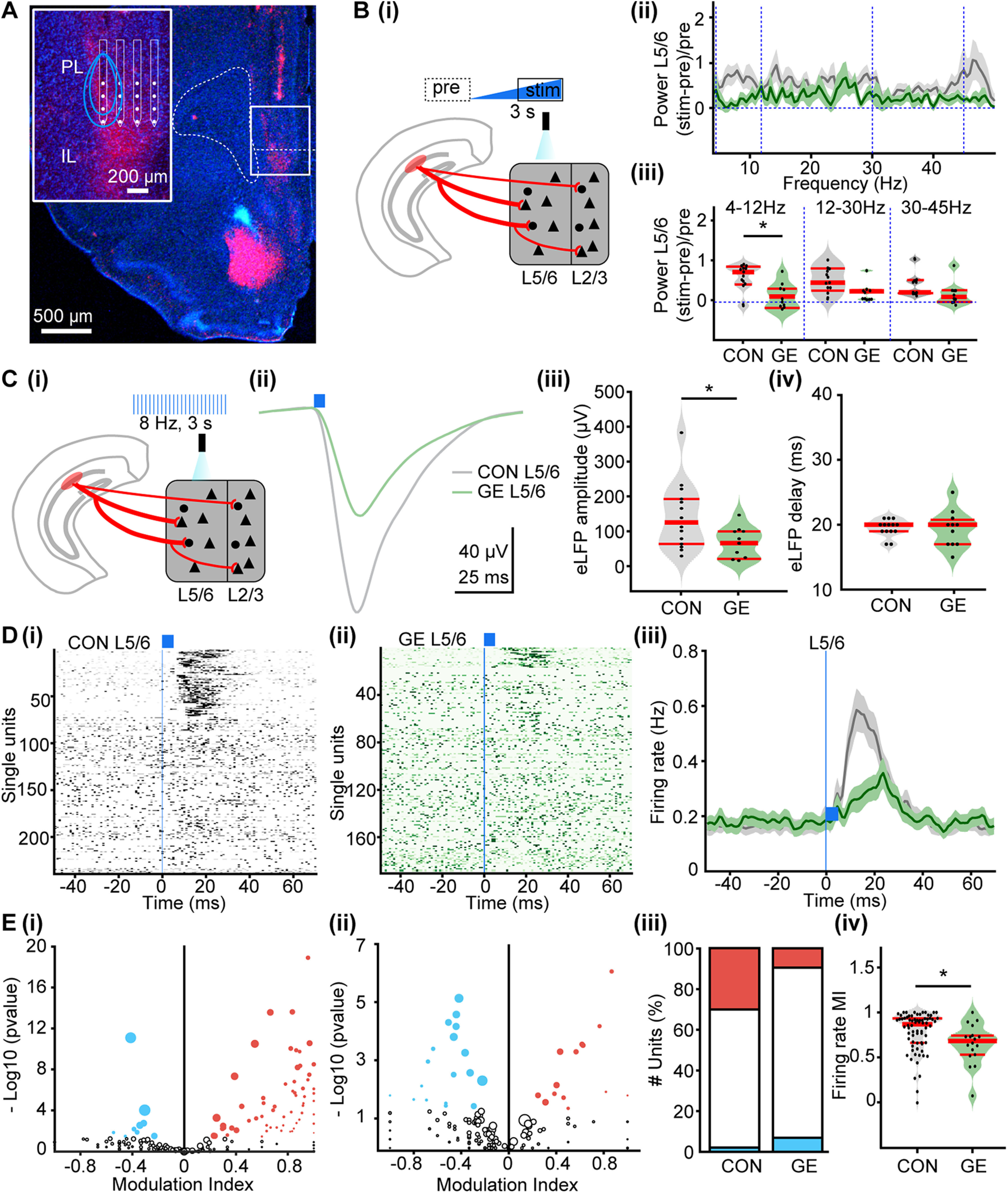
Oscillatory activity and neuronal firing in PL layer 5/6 after optogenetic activation of hippocampal terminals in neonatal CON and GE mice *in vivo*. ***A***, Digital photomontage reconstructing the location of a four-shank recording electrode in a DAPI-stained 100-μm-thick coronal section (blue) with hippocampal terminals expressing ChR2 (H134R; red) from a P9 mouse. Inset, Position of recording sites (white) over the PL layers displayed at higher magnification. Blue lines correspond to the iso-contour lines of light intensity (diameter 50 μm, numerical aperture 0.22, light parameters: 473 nm, 2 mW) for 5 and 10 mW/mm^2^. ***Bi***, Schematic of ramp light stimulation of hippocampal terminals in layer 5/6 of PL. ***ii***, Power of oscillatory activity in layer 5/6 during ramp stimulation of hippocampal terminals in layer 5/6, normalized to the activity 1.5 s before stimulation in CON (gray) and GE (green) mice. ***iii***, Violin plots displaying the oscillatory power averaged for different frequency bands (4–12, 12–30, 30–50 Hz) in response to ramp stimulation for all investigated CON and GE mice. ***Ci***, Schematic of pulses light stimulation of hippocampal terminals in layer 5/6 of PL. ***ii***, Averaged LFP traces recorded in layer 5/6 in response to light stimulation of HP terminals (blue bars) in CON (gray) and GE (green) mice. ***iii***, Violin plots showing the average amplitude of the maximum LFP response in layers 5/6 of CON and GE mice. ***iv***, Violin plots showing the average delay of the maximum LFP response in layers 5/6 of CON and GE mice. ***Di***, Raster plot depicting the firing of single PL cells in response to the pulse stimulation of hippocampal terminals in layer 5/6 of CON mice. ***ii***, Same as ***i***, for GE mice. ***iii***, Firing rate of all units in layer 5/6 around the pulse stimulation averaged for CON (gray) and GE (green) mice. ***Ei***, MI of spiking response of prefrontal single units to pulse stimulation in layer 5/6 of CON mice. MI > 0 indicates increased firing activity, whereas values < 0 correspond to decreased firing activity. ***ii***, Same as ***i***, for GE mice. ***iii***, Stacked bar plot showing the percentage of activated (red), unmodulated (white), and inhibited (blue) units after the pulse stimulation in layers 5/6 of CON and GE. ***iv***, Violin plots showing the MI of firing rate of all activated units in layers 5/6 of CON and GE. Single data points are represented as dots and the red horizontal bars in violin plots correspond to the median and the 25th and 75th percentiles; *p < 0.05.

To directly test this hypothesis, we investigated the impact of hippocampal inputs on the oscillatory entrainment of local circuits in the PL of CON and GE mice *in vivo*. For this, multisite extracellular recordings of LFP and multiunit activity (MUA) were performed in PL layer 5/6 and layer 2/3 of P8–P10 CON (*n* = 14) and GE mice (*n* = 11) before, during and after repetitive stimulation with ramp light stimuli or pulse trains (Fig. 4*A*). In line with our previous results, the used light intensity (0.75–2.5 mW) led to a temperature increase of max. 0.2°C, which is far below the local tissue heating that might interfere with neuronal spiking (Stujenske et al., 2015; Bitzenhofer et al., 2017). Ramp stimulation (3 s) significantly augmented theta band (4–12 Hz) oscillatory power in layer 5/6 of CON but not GE mice (0.807 ± 0.121 vs 0.218 ± 0.096, *F*_(1,23)_ = 3.365, *p* = 0.039; Fig. 4*B*; Table 4). Similarly, the magnitude of LFP response to light pulse trains (5-ms-long, 8-Hz, total duration of a train 3 s) significantly differed between the two groups (Fig. 4*C*). Activation of hippocampal terminals in layer 5/6 by pulsed light caused a large short-delay (∼19 ms) LFP depolarization that had a smaller amplitude in GE mice when compared with CON mice (144.9 ± 26.97 vs 67.0 ± 13.80 µV, *F*_(1,23)_ = 3.396, *p* = 0.024; Fig. 4*Ciii*; Table 4). Moreover, the firing of PL neurons changed after pulsed light stimulation (Fig. 4*D*). Analysis of SUA revealed that a prominent augmentation (311%) of firing rate occurred ∼13 ms after the stimulation in 72 out of 239 units (∼30.1%) recorded in layer 5/6 of CON mice. In GE mice, only 18 out of 189 units (∼9.5%) responded to light stimuli with a weaker (98.4%) and delayed (∼22 ms) firing rate increase (Fig. 4*D*,*E*). Analysis of MI of the firing rate of all activated units showed that the activated GE neurons fired significantly less when compared with CON (0.796 ± 0.023 vs 0.615 ± 0.054, *F*_(1,88)_ = 11.5007, *p* = 0.001; Fig. 4*Eiv*). These results indicate that not only hippocampal terminals target fewer prefrontal neurons in GE, but also their efficacy in boosting the firing rate is attenuated.

**Table 4.** Prefrontal activity patterns induced by light stimulation of hippocampal terminals at neonatal and prejuvenile age

			Neonatal			Prejuvenile		
			CON L5/6	GE L5/6	F values	CON L5/6	GE L5/6	F values
Layer 5/6 stimulation	Power (Stim-pre)/pre	4–12 Hz	0.807 ± 0.121	0.218 ± 0.096	*F*_(1,23)_ = 3.365 *p* = 0.039	0.071 ± 0.036	−0.02 ± 0.034	*F*_(1,26)_ = 1.716 *p* = 0.190
		12–30 Hz	0.701 ± 0.141	0.216 ± 0.0562	*F*_(1,23)_ = 1.779 *p* = 0.182	0.056 ± 0.027	0.042 ± 0.019	*F*_(1,26)_ = 0.114 *p* = 0.735
		30–45 Hz	0.383 ± 0.062	0.151 ± 0.061	*F*_(1,23)_ = 2.913 *p* = 0.0846	0.063 ± 0.043	0.079 ± 0.023	*F*_(1,26)_ = 0.179 *p* = 0.673
	evoked LFP	Amplitude (µV)	144.9 ± 26.97	67.0 ± 13.80	*F*_(1,23)_ = 3.396 *p* = 0.024	70.8 ± 7.88	63.1 ± 3.43	*F*_(1,26)_ = 0.007 *p* = 0.936
		Delay (ms)	19.3 ± 0.40	19.3 ± 0.89	*F*_(1,23)_ = 0.365 *p* = 0.552	17.5 ± 0.41	17.1 ± 0.57	*F*_(1,26)_ = 0.392 *p* = 0.537
Layer 2/3 stimulation	Power (Stim-pre)/pre	4–12 Hz	0.289 ± 0.109	0.106 ± 0.135	*F*_(1,21)_ = 2.314 *p* = 0.128	0.052 ± 0.029	0.0003 ± 0.031	*F*_(1,26)_ = 1.517 *p* = 0.218
		12–30 Hz	0.285 ± 0.103	0.272 ± 0.142	*F*_(1,21)_ = 0.314 *p* = 0.575	0.028 ± 0.028	0.036 ± 0.021	*F*_(1,26)_ = 0.070 *p* = 0.792
		30–45 Hz	0.231 ± 0.074	0.098 ± 0.066	*F*_(1,21)_ = 2.827 *p* = 0.093	0.044 ± 0.031	0.036 ± 0.030	*F*_(1,26)_ = 0.008 *p* = 0.930
	evoked LFP	Amplitude (µV)	132.3 ± 27.6	63.0 ± 17.94	*F*_(1,21)_ = 3.939 *p* = 0.047	58.9 ± 8.20	39.7 ± 4.83	*F*_(1,26)_ = 3.282 *p* = 0.07
		Delay (ms)	19.8 ± 0.48	20.2 ± 0.68	*F*_(1,21)_ = 0.013 *p* = 0.910	17.6 ± 0.32	17.2 ± 0.52	*F*_(1,26)_ = 0.910 *p* = 0.350

Data are shown as mean ± SEM. Significance was assessed using one-way ANOVA test followed by Bonferroni-corrected *post hoc* test. The listed *p* values correspond to comparisons CON L5/6 versus GE L5/6 and CON L2/3 versus GE L2/3.

**Figure 5.**
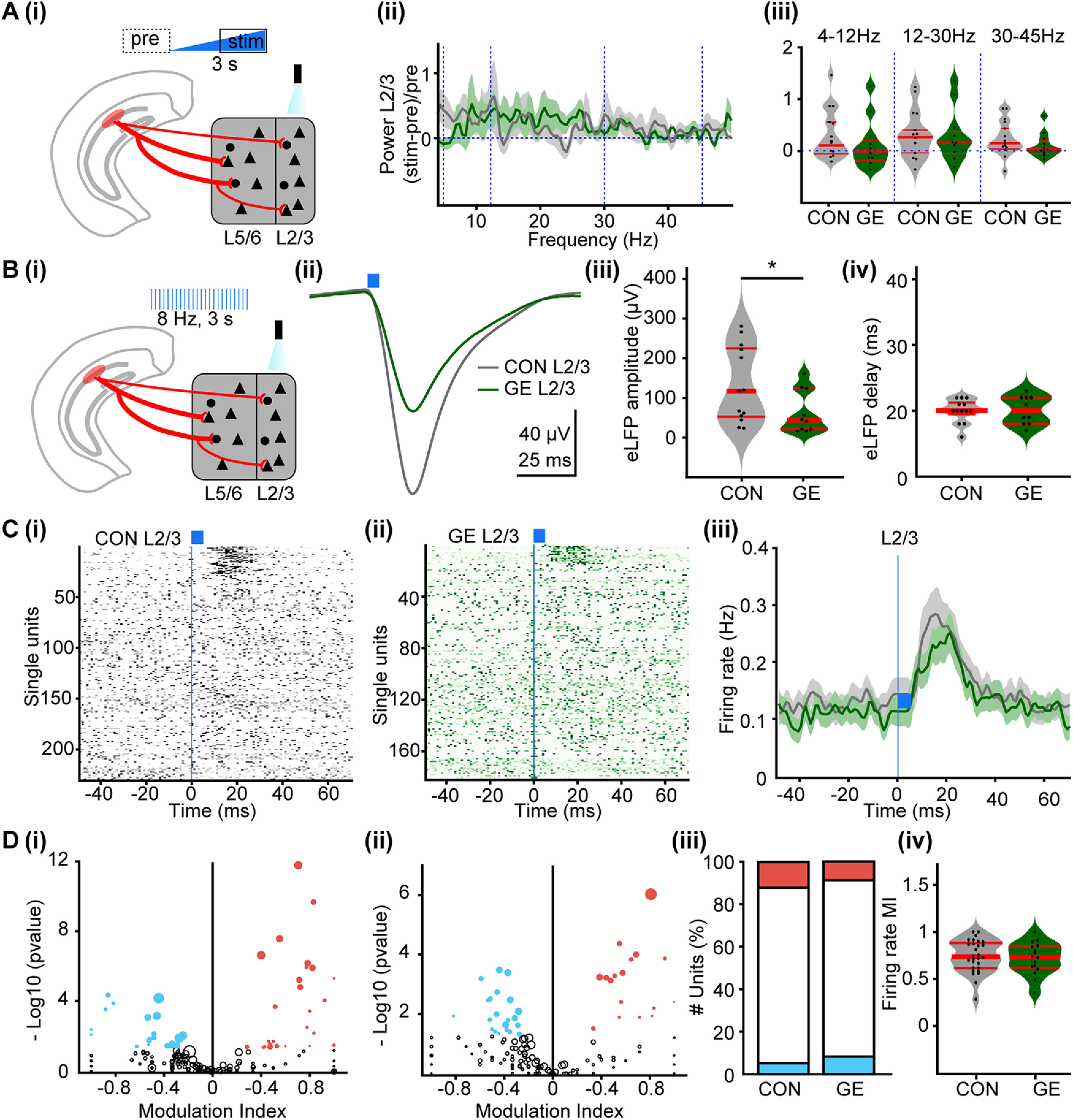
Oscillatory activity and neuronal firing in PL layer 2/3 after optogenetic activation of hippocampal terminals in neonatal CON and GE mice *in vivo*. ***Ai***, Schematic of ramp light stimulation of hippocampal axonal terminals in layer 2/3 of PL. ***ii***, Power of oscillatory activity in PL layer 2/3 during ramp stimulation of hippocampal terminals in layer 2/3, normalized to the activity 1.5 s before stimulation in CON (gray) and GE (green) mice. ***iii***, Violin plots displaying the oscillatory power averaged for different frequency bands (4–12, 12–30, 30–50 Hz) in response to ramp stimulation for all investigated CON and GE mice. ***Bi***, Schematic of pulses light stimulation of hippocampal terminals in layer 2/3 of PL. ***ii***, Averaged LFP response recorded in PL layer 2/3 in response to light stimulation (blue bars) of HP terminals in CON (gray) and GE (green) mice. ***iii***, Violin plots showing the average amplitude of the maximum LFP response evoked by light in layers 2/3 of CON and GE. ***iv***, Violin plots showing the average delay of the maximum LFP response evoked by light in layers 2/3 of CON and GE. ***Ci***, Raster plot depicting the firing of single PL cells in response to the pulse stimulation of hippocampal terminals in layer 2/3 of CON mice. ***ii***, Same as ***i***, for GE mice. ***iii***, Firing rate of all units in layer 2/3 around the pulse stimulation averaged for CON (gray) and GE (green) mice. ***Di***, MI of spiking response of prefrontal single units to pulse stimulation in layer 2/3 of CON mice. MI > 0 indicates increased firing activity, whereas values <0 correspond to decreased firing activity. ***ii***, Same as ***i***, for GE mice. ***iii***, Stacked bar plot showing the percentage of activated (red), unmodulated (white), and inhibited (blue) units after the pulse stimulation in layers 2/3 of CON and GE. ***iv***, Violin plots showing the MI of firing rate of all activated units in layers 2/3 of CON and GE. Single data points are represented as dots and the red horizontal bars in violin plots correspond to the median and the 25th and 75th percentiles; *p < 0.05.

In line with the fewer hippocampal axons targeting PL upper layers (Figs. 1*C*, 4*A*) even under physiological conditions, their activation with ramp light stimuli led to weak, if any, network effects in both CON (*n* = 13) and GE mice (*n* = 10; Fig. 5*A*; Table 4). However, the evoked LFP response had a lower amplitude in GE mice (132.3 ± 27.6 vs 63.0 ± 17.94 µV, *F*_(1,21)_ = 3.939, *p* = 0.047; Fig. 5*B*; Table 4). The firing rate and onset of light-induced firing were similar in all investigated mice (Fig. 5*C*). A smaller fraction of responsive units has been detected in GE (16 out of 18, ∼18.8%) when compared with CON (28 out of 230, ∼12.2%) mice (Fig. 5*Di–iii*). The firing rate MI of all activated units did not differ between CON and GE (0.763 ± 0.029 vs 0.750 ± 0.040, *F*_(1,42)_ = 0.079, *p* = 0.780; Fig. 5*Div*). In line with the data, we propose that the weaker hippocampal innervation of deep PL layers causes poor activation of targeted neurons, whereas similar effects are lacking for neurons in upper layers. However, the strong interlayer communication amplified within the densely packed upper layers (and possibly “contaminated” by volume conduction) might lead to disrupted network entrainment in both deep and upper layers, as reflected by the smaller amplitude of evoked LFP in GE mice.

These results indicate that, especially in layer 5/6, the hippocampal innervation has a weaker power to boost the firing and oscillatory activity in the PL of GE mice.

### Persistent dysfunction of hippocampal drive to PL cortex in prejuvenile dual-hit GE mice

Since previous studies showed major functional and behavioral deficits as a result of abnormal prefrontal-hippocampal communication in juvenile GE mice (Xu et al., 2019, 2021; Chini et al., 2020), it is likely that this dysfunction persists along with development. To test this hypothesis, we monitored the function of hippocampal innervation of PL in CON and GE mice at prejuvenile age.

**Figure 6.**
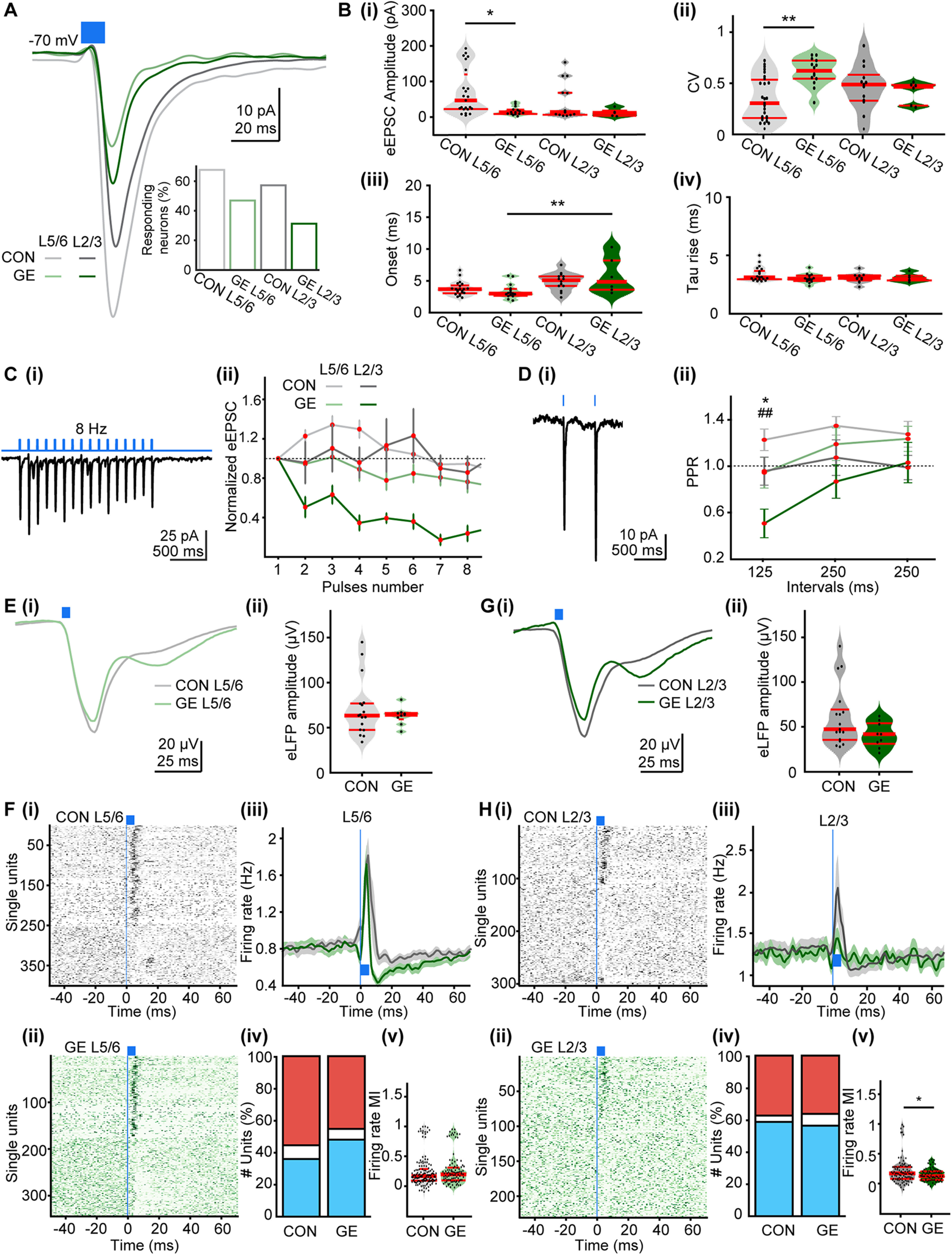
Responses of PL during optogenetic activation of hippocampal terminals in prejuvenile CON and GE mice *in vitro* and *in vivo*. ***A***, Averaged eEPSC (holding potential of –70 mV) evoked by light in layer 5/6 (*n* = 23) and layer 2/3 (*n* = 15) neurons from prejuvenile CON mice as well as in pyramidal neurons in layer 5/6 (*n* = 12) and layer 2/3 (*n* = 6) from prejuvenile GE mice. Blue bar corresponds to 10-ms light stimulation. Inset, Bar diagram of the percentage of responsive pyramidal neurons in different groups. ***B***, Violin plots showing the (***i***) amplitudes, (***ii***) CV of amplitudes, (***iii***) synaptic delay, and (***iv***) rise tau of eEPSCs averaged for all prefrontal neurons in CON and GE mice. ***Ci***, Representative current response to pulsed light (8 Hz; blue) of a layer 5/6 pyramidal neuron from a P21 CON mouse. ***ii***, Plot of eEPSC amplitude (normalized to the first EPSC amplitude) in response to 8-Hz stimulation averaged for all prefrontal neurons in CON and GE mice. ***Di***, Representative response to light stimuli (500-ms interstimulus interval) of a layer 5/6 pyramidal neuron from a P21 CON mouse. ***ii***, Plot of PPR at 125-, 250-, 500-ms interstimulus intervals averaged for all prefrontal neurons in CON and GE mice; * for comparison of layer 5/6, ## for comparison of layer 2/3. ***Ei***, Averaged LFP response recorded in PL layer 5/6 in response to light stimulation (blue bars) of HP terminals in CON (gray) and GE (green) mice. ***ii***, Violin plots showing the average amplitude of the maximum LFP response evoked by light in layer 5/6 of CON and GE mice. ***Fi***, Raster plot depicting the firing of single PL cells in response to pulse stimulation of hippocampal terminals in layer 5/6 of CON mice. ***ii***, Same as ***i***, for GE mice. ***iii***, Firing rate of all units in layer 5/6 around the pulse stimulation averaged for CON (gray) and GE (green) mice. ***iv***, Stacked bar plot showing the percentage of activated (red), unmodulated (white), and inhibited (blue) units after pulse stimulation of PL layer 5/6 of CON and GE mice. ***v***, Violin plots showing the MI of firing rate of all activated units in layer 5/6 of CON and GE mice. ***G***, Same as in ***E***, but for the stimulation in layer 2/3 of PL. ***H***, Same as in ***F***, but for spike response of single prefrontal cells to pulse stimulation of hippocampal terminals in layer 2/3. Single data points are represented as dots and the red horizontal bars in violin plots correspond to the median and the 25th and 75th percentiles; *p < 0.05, **p < 0.01, ^##^*p* < 0.01.

First, the function of hippocampal projections in PL was assessed *in vitro*. Similar to the results obtained from coronal slices including the PL from neonatal mice, light stimulation (10 ms, 473 nm) of hippocampal inputs evoked robust EPSCs in prefrontal neurons from all investigated prejuvenile mice (Fig. 6*A*). However, the fraction of responsive neurons was larger in CON (layer 5/6: 67.65%; layer 2/3: 57.14%) when compared with GE mice (layer 5/6: 46.88%; layer 2/3: 31.25%; Table 3). The light-induced synaptic inputs had faster kinetics when compared with the currents recorded in the neonatal PL in all investigated prejuvenile mice (Fig. 6*Biii,iv*; Table 3). The amplitude of the eEPSCs recorded in layer 5/6 was significantly smaller in GE (70.1 ± 13.74 vs 19.7 ± 4.39 pA, *p* = 0.0354; Fig. 6*Bi*) and had a higher variability when compared with CON mice (Fig. 6*Bii*; Table 3). In contrast, the eEPSCs recorded from layer 2/3 neurons were similar in prejuvenile CON and GE mice. Repetitive stimulation (8 Hz, 10 ms) of hippocampal inputs evoked sustained EPSCs with different response patterns in CON and GE neurons. In contrast to the prominent depression of inputs in all neonatal neurons, a slight depression was detected for layer 2/3 neurons of GE mice, whereas the eEPSCs in layer 5/6 were either facilitated or unchanged (Fig. 6*C*). However, PPR for layer 2/3 decreased in GE mice and showed a clear depression over higher frequencies. Moreover, there was a significant difference in the value of PPR between CON and GE when light was conducted at 125-ms intervals, but not at 250- and 500-ms intervals (Fig. 6*Dii*; Table 3). This means the STP of hippocampal inputs was comparable in response to low frequency stimulation in CON and GE, but differs for high frequency stimulation. Taken together, these results indicate that the dysfunction of hippocampal innervation persists at prejuvenile age in GE mice, yet it appears less pronounced than the deficits reported for neonatal stage.

Second, we performed multisite extracellular recordings of LFP and MUA combined with the optogenetic stimulation of hippocampal terminals in prejuvenile CON (*n* = 17) and GE mice (*n* = 9) *in vivo* (Fig. 6*E–H*). We used similar stimulation protocols as described for neonatal animals. Ramp light stimulation of hippocampal projections targeting PL layers 5/6 and 2/3 had a minor, if any, effect on the power of network oscillation in CON and GE mice (Table 4). The pulsed light evoked a strong bi-phasic LFP response (Fig. 6*Ei*,*Gi*) with comparable amplitude in all investigated mice (Fig. 6*Eii*,*Gii*; Table 4). The overall PL firing was augmented on light stimuli, yet the number of responsive units was lower in layer 5/6 of GE mice (155 out of 342, ∼45.3%) when compared with CON mice (221 out of 398, ∼55.5%; Fig. 6*Fiv*). The firing rate MI of all activated units did not differ between CON and GE (0.225 ± 0.023 vs 0.239 ± 0.028, *F*_(1,374)_ = 0.1372, *p* = 0.712; Fig. 6*Fv*). When the light activated the hippocampal axonal terminals in layer 2/3, the firing rate strongly augmented in CON but much weaker in GE mice (Fig. 6*H*). However, the number of activated units was comparable in the two groups of prejuvenile mice (CON: 121 out of 327, ∼38.8%; GE: 86 out of 230 units, ∼37.3%; Fig. 6*Hiv*). The firing rate MI of all activated units was significant lower in GE when compare to CON (0.178 ± 0.024 vs 0.121 ± 0.013, *F*_(1,206)_ = 3.881, *p* = 0.049; Fig. 6*Hv*).

Thus, the functional disruption of hippocampal drive to the PL in dual-hit GE mice persists throughout development, although the magnitude and patterns of dysfunction differ from those identified at neonatal age.

## Discussion

Many decades ago, disturbed interactions between HP and PFC has been proposed as a core aspect of pathophysiology in psychiatric disorders. Especially in schizophrenia, the prefrontal-hippocampal impairment might link the neurodevelopmental dysfunction and later behavioral deficits (Weinberger, 1987). However, until recently, the experimental evidence for abnormal disease-related prefrontal-hippocampal communication during development was missing. We previously capitalized on *in vivo* recording and manipulation techniques in mouse models of disease and showed that the development of local circuits in both PFC and HP are profoundly impaired when genetic and environmental stressors converge to mimic the psychiatric risk (Xu et al., 2019, 2021; Chini et al., 2020). Moreover, the excitatory drive from the HP to PFC is weaker in these disease models (Hartung et al., 2016; Oberlander et al., 2019). In the present study, we monitor the structure and function of prefrontal-hippocampal connectivity in control and GE mice. We show that in GE mice (1) the sparser axonal projections from HP to PL act as substrate of diminished HP-PFC communication throughout postnatal development; (2) presynaptic abnormality of hippocampal terminals and their poorer efficiency in activating the PL cause miswiring of long-range connectivity; and (3) the deficits of hippocampal projections persist, yet at a lower magnitude, until prejuvenile age.

A wealth of studies documented the schizophrenia-characteristic dysconnectivity between HP and PFC in chronic patients, first-episode patients as well as high-risk individuals during cognitive tasks (Meyer-Lindenberg et al., 2005; Benetti et al., 2009; Wolf et al., 2009). The weaker driving force from the HP to PFC has been replicated in different animal models of disease at adult age (Dickerson et al., 2010; Sigurdsson et al., 2010; Mukai et al., 2015). Three possible sources of disconnection have been identified. First, the excitatory drive from the HP is decreased because of cellular dysfunction and altered morphologic features of CA1 pyramidal neurons. Postmortem histology in schizophrenia patients and mouse models as well as monitoring of neuronal and network activity in HP *in vivo* and *in vitro* confirmed this hypothesis (Harrison and Weinberger, 2005; Meyer-Lindenberg, 2010; Marissal et al., 2018). Second, abnormal structure and function of both prefrontal pyramidal neurons and interneurons might hamper the normal communication between HP and PFC (Benchenane et al., 2010; Mukai et al., 2015; Sauer et al., 2015; Abbas et al., 2018). Third, decreased connectivity between the two brain areas might serve as a substrate of the decoupling monitored by decreased synchrony between HP and PFC (Meyer-Lindenberg et al., 2005; Cohen, 2011; Mukai et al., 2015).

The developmental dysconnectivity between HP and PFC was observed in several animal models of psychiatric disorders that mirror distinct aspects of the disease (Oberlander et al., 2019). In particular, mice that combine the genetic deficits with the action of environmental stressors to mimic the psychiatric risk showed disconnection of PFC and HP toward the end of the first postnatal week (Hartung et al., 2016; Oberlander et al., 2019), a developmental stage that corresponds to the second-third gestational trimester in humans (Clancy et al., 2001). However, in contrast to the previously reported dysfunction in adult mice (Kvajo et al., 2008, 2011; Dickerson et al., 2010), DISC1 suppression or MIA alone (single-hit models) had no impact on the neuronal and network function at neonatal age. In dual-hit GE mice, the structure and function of PFC and HP were compromised at neonatal age and the deficits persist, yet sometimes at a lower magnitude, throughout the entire development (Xu et al., 2019, 2021; Chini et al., 2020). These observations support the concept that convergence of genetic and environmental risk factors advances the neuropathology of disease and might cause severer deficits (Uher, 2014). In the present study, we complemented these data and provided experimental evidence for the early prefrontal-hippocampal disconnection.

Monitoring of hippocampal projections by different methods revealed the sparser targeting of PFC. The role of DISC1 in dendritic and axonal development is well documented (Morris et al., 2003; Ozeki et al., 2003; Shen et al., 2008; Kvajo et al., 2011). Mutations in Disc1 lead to alterations in neuronal architecture and cognition (Kvajo et al., 2008, 2011; Crabtree et al., 2017), that have been reported for schizophrenia, bipolar disorder, and major depression (Millar et al., 2000; Blackwood et al., 2001). Given the ability of DISC1 to interact with proteins that bind to microtubules and associated complexes, thus regulating cytoskeleton dynamics (Morris et al., 2003; Ozeki et al., 2003; Brandon et al., 2005; Wang and Brandon, 2011), it is not surprising that the long-range axonal projections from HP to PFC are significantly reduced in GE mice. Reduced integrity and anatomic abnormalities in the fornix, the fiber bundle that connects the HP with neocortical areas including the PFC, have been observed in schizophrenia patients. Moreover, hippocampal projections form fewer branches in the PFC of mouse models and has been proposed as an anatomic substrate of prefrontal-hippocampal synchrony deficits (Zhou et al., 2008; Mukai et al., 2015). In line with the structural change, the diminishment of excitatory drive toward PFC neurons was observed. Hippocampal terminals targeted fewer prefrontal neurons in GE mice and their efficacy in boosting the firing of prefrontal neurons was much weaker.

Besides the decreased axonal density, multiple presynaptic alterations of hippocampal inputs were found in dual-hit GE mice. The observed AP widening might lead to altered short-term synaptic plasticity by increasing the initial probability of presynaptic release and shifting the presynaptic STP toward depression (Abbott and Regehr, 2004). PPR directly relates to presynaptic release probability, yet also re-uptake mechanisms and modulation of neurotransmitter vesicle fusion (Glasgow et al., 2019). Furthermore, our observation that the differences between neonatal CON and GE in short-term depression paradigms were most obvious at lower stimulation frequency (500-ms interval) than at higher stimulus frequency (125-ms interval) supports the impact of wider APs in the HP for neurotransmitter release in PFC. It has been shown that the presynaptic characteristics are not fixed throughout development. The depression contributes less to synaptic dynamics, whereas facilitation becomes more prominent (Reyes and Sakmann, 1999; Dittman et al., 2000). At prejuvenile age, short-term facilitation of hippocampal terminals on prefrontal neurons was observed in CON, supporting the synaptic enhancement during development. However, in GE mice high frequency depression was observed in layer 2/3 neurons of PL. The underlying mechanisms might be presynaptic deregulation of the synaptic vesicle recycling and the release of neurotransmitters (Flores et al., 2011; Tang et al., 2016) or postsynaptic receptor desensitization that make the target neurons less sensitive to neurotransmitter (Zucker and Regehr, 2002). From neonatal to prejuvenile age, the *in vivo* LFP response to light stimulation of hippocampal terminals was weaker at prejuvenile age, which might relate to a more mature network balanced by excitation-inhibition. However, the response becomes faster as shown by the shorter onset of eEPSCs and faster firing of prefrontal neurons. These changes are less evident in GE mice. Overall, in addition to the reduced hippocampal innervation and ability in entraining PL activity, alterations in synaptic plasticity reduce the efficiency of these projections. Altogether, these processes may cumulatively impinge on the structure and function of hippocampal-prefrontal network.

The results of light stimulation in CON mice provide first insights into the mechanisms of how hippocampal inputs shape the prefrontal excitation-inhibition throughout the development. At neonatal age, few neurons reduced their firing rate after pulsed light stimulation, whereas their number significantly augmented at prejuvenile age. The decreased firing rates might arise from the feed-forward inhibition of the interneurons that are directly targeted by hippocampal terminals, or from the activation of interneurons directly connected to the light-activated pyramidal neurons. Multiple mechanisms, such as more interneurons are recruited by the hippocampal innervation, the synaptic strength on interneurons increase along with the development, or the interaction between pyramidal neurons and interneurons change with age, might underlie these observations. Our results showed that the depression-to-facilitation shift of hippocampal input on layer 2/3 PL neurons is disrupted in prejuvenile GE mice. Excitation-inhibition imbalance in PL might underlie numerous neurologic and behavioral abnormalities found in GE mice.

The present results add experimental evidence for the developmental miswiring of prefrontal-hippocampal networks in psychiatric disorders. The profound dysfunction of these networks already takes place at early stages of development. The sparse and less efficient projections from CA1 area to the PFC do not optimally entrain the prefrontal networks in oscillatory rhythms. Together with the local synaptic deficits in both areas, the weaker connectivity causes an abnormal communication and information processing that, despite partial compensation at prejuvenile age, might be vulnerable to environmental stressors or age-related changes of neuromodulatory systems (e.g., dopamine; Arnsten et al., 2012; McEwen and Morrison, 2013; Klune et al., 2021). By these means, the early disconnection between PFC and HP might have a long-lasting impact on memory and executive processing (Hartung et al., 2016; Xu et al., 2019, 2021; Chini et al., 2020). While these data from animal models of disease help to identify possible “hubs” of miswiring early in life, future investigations need to explore the clinical validity of developmental mechanisms of mental disorders.
